# Uniform Finite Element Error Estimates with Power-Type Asymptotic Constants for Unsteady Navier–Stokes Equations

**DOI:** 10.3390/e24070948

**Published:** 2022-07-07

**Authors:** Cong Xie, Kun Wang

**Affiliations:** 1 College of Mathematics and Systems Science, Xinjiang University, Urumqi 830046, China; xiecong121@163.com; 2 School of Mathematics and Computer Application Technology, Jining University, Jining 273155, China; 3 College of Mathematics and Statistics, Chongqing University, Chongqing 401331, China

**Keywords:** Navier–Stokes equations, power-type asymptotic constant, long-time stability, finite element method, error estimate

## Abstract

Uniform error estimates with power-type asymptotic constants of the finite element method for the unsteady Navier–Stokes equations are deduced in this paper. By introducing an iterative scheme and studying its convergence, we firstly derive that the solution of the Navier–Stokes equations is bounded by power-type constants, where we avoid applying the Gronwall lemma, which generates exponential-type factors. Then, the technique is extended to the error estimate of the long-time finite element approximation. The analyses show that, under some assumptions on the given data, the asymptotic constants in the finite element error estimates for the unsteady Navier–Stokes equations are uniformly power functions with respect to the initial data, the viscosity, and the body force for all time t>0. Finally, some numerical examples are shown to verify the theoretical predictions.

## 1. Introduction

We study the long-time finite element error estimates for the time-dependent Navier–Stokes equations (NSE)

(1) $$u_{t}-\nu \Delta u+\left(u\cdot \nabla \right)u+\nabla p=f,\;\;\;\mathrm{div}\;u=0,\;\;\forall \left(x,t\right)\in \Omega \times \left(0,+\infty \right),$$

(2) $$u\left(x,0\right)=u_{0}{\left(x\right),\;\;\forall x\in \Omega ,\;\;\;\;\;\;\;\;\;u\left(x,t\right)|}_{\partial \Omega }=0,\;\;\forall t\in \left[0,+\infty \right),$$

where $u=u\left(x,t\right)={\left(u_{1}\left(x_{1},x_{2},t\right),u_{2}\left(x_{1},x_{2},t\right)\right)}^{T}$ is the velocity; p=p(x) is the pressure; ν>0 is the viscosity; $f=f\left(x\right)={\left(f_{1}\left(x_{1},x_{2},t\right),f_{2}\left(x_{1},x_{2},t\right)\right)}^{T}$ is the prescribed body force; $u_{(}x)$ is the initial data satisfying $\mathrm{div}\;u_{0}=0$; and Ω is a bounded domain in $R^{2}$, which has a Lipschitz continuous boundary ∂Ω and satisfies the additional condition stated in (**A1**) below.

Problems (1)–(2) form the famous incompressible Newtonian fluid model. Many efficient numerical schemes have been developed to approximate this problem. As a classical one, the finite element approximation for this problem has been widely investigated. In this field, investigating the long-time stability and error of the finite element method is a very popular topic, which has practical interests in engineering, weather prediction, and so on. For the finite element semidiscrete scheme of the Navier–Stokes equations, Heywood and Rannacher [1,2] analyzed uniform error estimates by assuming that the exact solution is exponentially stable. Further investigation was conducted in [3] for the stabilized finite element method, in which the authors avoided the assumption on the exact solution but used an exponential-type factor $e^{\tilde{c}t}$ in the asymptotic coefficient $(\tilde{c}$ is a general positive constant and t∈[0,T], with *T* being a finite time satisfying that if *t* is not in the neighborhood of +∞, then *t* must be in [0,T]). Some related works continued the investigation in [4,5,6,7]. For the long-time stability of fully discrete schemes, Simo and Armero [8] proved that several time integration schemes are unconditional stable for the long-time approximation. Furthermore, He and Li [9,10] and Tone and Wirosoetisno [11] studied the implicit Euler scheme, Tone [12] deduced the Crank/Nicolson scheme, Breckling and Shields [13] investigated the linearly extrapolated second-order scheme, and Ngondiep [14] analyzed a two-level hybrid method for the time-dependent Navier–Stokes equations. Other researches on this topic can be also found in [15,16,17,18]. On the other hand, other forms of the problem, including the vorticity-stream form and the rotation form, have been studied. By rewriting (1) and (2) to the vorticity-stream form, Gottlieb et al. [19] considered the implicit–explicit scheme, and Cheng and Wang [20] investigated multistep high-order schemes. The accuracy of the rotation form was studied by Layton et al. in [21]; a new EMA-conserving (EMAC) formulation, which conserved energy, momentum, angular momentum, was presented by Charnyi et al. in [22]; and a high-order pressure-robust method for the rotation form was developed by Yang et al. in [23]. In all of these analysis, the Gronwall lemma was used in deriving the stability in the energy norm for the schemes, which led to exponential-type asymptotic constants with respect to the given data. Recently, via transforming the primitive Equations (1) and (2) to the velocity–vorticity formulation, Heister et al. [24] deduced the long-time stability with power-type constants for the backward Euler and BDF2 schemes. However, to the best of our knowledge, there is no result on the error estimate with power-type asymptotic constants in the literature.

When analyzing the stability and error estimate for a nonlinear problem, the Gronwall lemma is usually used and an exponential-type factor will appear in the asymptotic constant, which is virtually meaningless when the given data (such as the time) are large. Although the Gronwall lemma is avoided by using the velocity–vorticity method in the fully discrete scheme (see [24]), it is still necessary for the finite element semidiscrete method according to the procedure in the literature. The reason is that the fully discrete scheme in the velocity–vorticity form can decouple the nonlinear term of the problem, but this is not true when deducing the error estimate. In this paper, we firstly prove the stability with power-type asymptotic constants of the finite element semidiscrete method for a linearized auxiliary problem. Then, we construct an iterative scheme for the nonlinear Equations (1) and (2) and extend the stability results for the linearized auxiliary problem to this iterative scheme by applying the inductive method. Under some assumptions on the given data, we confirm that the iterative sequences converge to the solution of the Navier–Stokes Equations (1) and (2). Thus, the stability for the iterative scheme also holds for the unsteady Navier–Stokes equations. Since the Gronwall lemma is avoided in our analysis, the generated asymptotic constants in these stabilities are uniformly power functions with respect to the viscosity, the initial data, and the body force. Although this iterative approximation methodology is used to derive the viscosity explicit estimate in our recent work [25], the analysis has focused on a finite time interval and contains an asymptotic constant $\tilde{c}t^{\alpha }\left(\alpha >0\right)$, which is also meaningless when the time attends to infinity. In this paper, by utilizing a weighted $L^{2}-$norm in the time, we derive the following results: if the given data satisfies

$$\frac{N\kappa_{2}}{\nu }<1,$$

it holds that

$$\begin{array}{cc} {\;||u||}_{L^{\infty }\left(0,+\infty ;L^{2}\left(\Omega \right)\right)}^{2}+{\nu ||u||}_{L^{2,\nu }\left(0,+\infty ;H^{1}\left(\Omega \right)\right)}^{2} & \leq e^{-\nu \lambda_{1}t}{\left|u_{0}\right|}^{2}+\frac{2C_{f}^{2}}{\nu^{2}\lambda_{1}}:=\kappa_{1}^{2},\\ {\;||u||}_{L^{\infty }\left(0,+\infty ;H^{1}\left(\Omega \right)\right)}^{2}+{\nu ||u||}_{L^{2,\nu }\left(0,+\infty ;H^{2}\left(\Omega \right)\right)}^{2} & \leq e^{-\nu \lambda_{1}t}\left|\right|u_{0}{\left|\right|}^{2}+\frac{C_{f}^{2}}{\nu^{2}\lambda_{1}}+5\kappa_{1}^{2}:=\kappa_{2}^{2},\\ \;||u_{t}{\left|\right|}_{L^{2,\nu }\left(0,+\infty ;L^{2}\left(\Omega \right)\right)}^{2}+{\left||p|\right|}_{L^{2,\nu }\left(0,+\infty ;L^{2}\left(\Omega \right)\right)}^{2} & \leq 2\left(\nu \kappa_{2}^{2}+\frac{C_{f}^{2}}{\nu^{2}\lambda_{1}}+\nu \kappa_{1}\kappa_{2}\right):=\kappa_{3}^{2},\\ \;||u_{t}{\left|\right|}_{L^{\infty }\left(0,+\infty ;L^{2}\left(\Omega \right)\right)}^{2}+\nu ||u_{t}{\left|\right|}_{L^{2,\nu }\left(0,+\infty ;H^{1}\left(\Omega \right)\right)}^{2} & \leq e^{-\nu \lambda_{1}t}{\tilde{C}}_{0}^{2}+\left(\nu +\nu \lambda_{1}+1\right)\kappa_{3}^{2}+\nu {\tilde{C}}_{f}^{2}:=\kappa_{4}^{2},\\ \nu^{2}{\left||u|\right|}_{L^{\infty }\left(0,+\infty ;H^{2}\left(\Omega \right)\right)}^{2}+{\left||p|\right|}_{L^{\infty }\left(0,+\infty ;H^{1}\left(\Omega \right)\right)}^{2} & \leq \nu^{2}\kappa_{1}^{2}+2\kappa_{4}^{2}, \end{array}$$

where *N* is a constant depending on Ω that will be determined in Section 2; $\kappa_{i}>0\;\left(i=1,\cdots ,4\right)$ are uniformly bounded with respect to the time *t*; $L^{2,\nu }\left(0,+\infty ,{\left(H^{i}\right)}^{d}\right)=\left\{u|e^{-\nu \lambda_{1}t}\int_{0}^{t}e^{\nu \lambda_{1}s}{\left||u|\right|}_{i}^{2}ds\leq \infty \right\}$; $\lambda_{1}$ is the minimal eigenvalue of the Laplace operator −Δ; and $C_{f}$ and ${\tilde{C}}_{0}$ are polynomials of the given data, which will be determined in Section 3. Moreover, according to the fixed-point theorem, we derive the long-time finite element error estimates as follows:

$$\begin{array}{cc} \;||u-u_{h}{\left|\right|}_{L^{\infty }\left(0,+\infty ;L^{2}\left(\Omega \right)\right)}^{2} & \leq \tilde{\kappa }h^{2},\\ \;\tau \left(t\right)||u-u_{h}{\left|\right|}_{L^{\infty }\left(0,+\infty ;H^{1}\left(\Omega \right)\right)}^{2} & \leq \tilde{\kappa }h^{2},\\ \tau^{2}\left(t\right)||p-p_{h}{\left|\right|}_{L^{\infty }\left(0,+\infty ;L^{2}\left(\Omega \right)\right)}^{2} & \leq \tilde{\kappa }h^{2}, \end{array}$$

where τ(t)=min{1,t} and $\tilde{\kappa }$ is a power function with respect to $u_{0},\;\nu$, and *f*, which may take different values at different occurrences.

The remainder of this paper is organized as follows: We introduce some functional settings for problems (1)–(2) in Section 2. Then, by investigating an auxiliary problem, we prove the stability with power-type asymptotic constants for the Navier–Stokes equations in Section 3. In Section 4, we extend the analysis technique to the error estimate. Some numerical examples are given to confirm the theoretical analysis in Section 5. Finally, conclusions are provided in Section 6.

## 2. Functional Setting

Before proceeding the analysis, we introduce the following functional settings:

$$X=H_{0}^{1}{\left(\Omega \right)}^{2},\;\;Y=L^{2}{\left(\Omega \right)}^{2},\;\;M=L_{0}^{2}\left(\Omega \right)=\left\{q\in L^{2}\left(\Omega \right);\int_{\Omega }qdx=0\right\}.$$

Denote by (·,·) and |·| the inner product and norm of $L^{2}\left(\Omega \right)$ or ${\left(L^{2}\left(\Omega \right)\right)}^{2}$, the usual scalar product ((u,v))=(∇u,∇v) and norm $\left||u|\right|={\left(\left(u,u\right)\right)}^{1/2}$ of $H_{0}^{1}\left(\Omega \right)$ or *X*, and by $\left|\right|\cdot {\left|\right|}_{i}$ the norm of the Sobolev space $H^{i}\left(\Omega \right)$ or ${\left(H^{i}\left(\Omega \right)\right)}^{2}$ for $i=0,1,2(||\cdot |{|}_{0}=|\cdot |).$ Moreover, let *H* and *V* be the closed subsets of *Y* and *X*, respectively, which are given by

$$H=\left\{v\in Y;\mathrm{div}\;v=0,v\cdot n{|}_{\partial \Omega }=0\right\},\;\;\;\;\;\;V=\left\{v\in X;\mathrm{div}\;v=0\right\}.$$

The Stokes operator is denoted by A=−PΔ, where *P* is the $L^{2}-$orthogonal projection of *Y* onto *H*.

Additionally, we need some assumptions on the domain Ω as that provided in [26]:

**(A1)**. Assume that Ω is smooth enough and $g\in L^{2}{\left(\Omega \right)}^{2}$ so that the unique solution (v,q)∈X×M of the steady Stokes problem

$${-\Delta v+\nabla q=g,\;\;\mathrm{div}\;v=0\;\;\mathrm{in}\;\Omega ,\;\;v|}_{\partial \Omega }=0$$

exists and satisfies

$${\left||v|\right|}_{2}+{\left||q|\right|}_{1}\leq c\left|g\right|.$$

Hereafter, *c* is a general positive constant independent of $u_{0},\nu ,f,t$ but depending on the domain Ω, which may take different values at different occurrences.

**(A1)** implies that

(3) $${\left||v|\right|}_{-1}^{2}\leq \lambda_{1}^{-1}{\left|v\right|}^{2}{,\;\;\;|v|}^{2}\leq \lambda_{1}^{-1}{\left||v|\right|}^{2}\;\;\;\forall v\in X,$$

(4) $${\left||v|\right|}^{2}\leq \lambda_{1}^{-1}{\left|Av\right|}^{2}{,\;\;\;||v||}_{2}^{2}\leq c{\left|Av\right|}^{2}\;\;\;\forall v\in D\left(A\right)={\left(H^{2}\left(\Omega \right)\right)}^{2}\cap V,$$

where $\lambda_{1}$ is the minimal eigenvalue of the Laplace operator −Δ. Moreover, some assumptions on the initial data and the body force for problems (1) and (2) are necessary.

**(A2).** There exist $C_{0}$ and $C_{f}$ such that the initial velocity $u_{0}\left(x\right)$ and the body force f(x,t) satisfy

$$u_{0}\in D\left(A\right),\;\mathrm{with}\;|Au_{0}|\leq C_{0},\;\;\;\;\underset{t\geq 0}{\sup}{\left(||f\left(t\right)|\right|}_{-1}+\left|f\left(t\right)\right|+{\left||f\left(t\right)|\right|}_{1}+|f_{t}\left(t\right)\left|\right)\leq C_{f}.$$

As usual, we define the continuous bilinear forms a(·,·) on X×X and d(·,·) on X×M, respectively, by

a(u,v)=((u,v))   ∀u,v∈X,   d(v,q)=(q,div v)   ∀v∈X,q∈M,

and the trilinear form b(·,·,·) on X×X×X by

$$\begin{array}{cc} \;b(u,v,w) & =\left(\left(u\cdot \nabla \right)v,w\right)+\frac{1}{2}\left(\left(\nabla \cdot u\right)v,w\right)\\ & =\frac{1}{2}\left(\left(u\cdot \nabla \right)v,w\right)-\frac{1}{2}\left(\left(\nabla \cdot u\right)w,v\right)\;\;\;\forall u,v,w\in X. \end{array}$$

It is well-known that the bilinear form a(·,·) defined above is continuous and coercive on X×X; further, for d(·,·), there exists a positive constant β>0 such that (see [1,26,27])

(5) $$\beta |q|\leq \underset{v\in X,v\neq 0}{\sup}\frac{\left|d(u,p)\right|}{\left||v|\right|}\;\;\;\;\forall q\in M.$$

For the trilinear form b(·,·,·), we have (see [26,27])

(6) b(u,v,w)=−b(u,w,v)   ∀u,v,w∈X,

(7) $$\left|b\left(u,v,w\right)\right|\leq {N|u|}^{1/2}{\left||u|\right|}^{1/2}{\left||v||\;|w\right|}^{1/2}{\left||w|\right|}^{1/2}\;\;\;\forall u,v,w\in X,$$

(8) |b(u,v,w)|≤N||u|| ||v|| ||w||   ∀u,v,w∈X,

(9) |b(u,v,w)|≤N|u| ||v|| |Aw|   ∀u,v∈X,w∈D(A),

(10) $$\left|b\left(u,v,w\right)\right|\leq {N|u|}^{1/2}{\left|Au\right|}^{1/2}||v||\;|w|\;\;\;\forall u\in D\left(A\right),v,w\in X,$$

(11) $$\left|b\left(u,v,w\right)\right|\leq {N|u|}^{1/2}{\left||u|\right|}^{1/2}{\left||v|\right|}^{1/2}{\left|Av\right|}^{1/2}\left|w\right|\;\;\;\forall u,w\in X,v\in D\left(A\right),$$

(12) $$\left|b\left(u,v,w\right)\right|\leq {N||u||\;||v||}^{1/2}{\left|Av\right|}^{1/2}\left|w\right|\;\;\;\forall u,w\in X,v\in D\left(A\right),$$

(13) $$\left|b\left(u,v,w\right)\right|\leq {N||u||\;||v||\;|w|}^{1/2}{\left||w|\right|}^{1/2}\;\;\;\forall u,v,w\in X.$$

With the above notations, the variational formulation of the time-dependent Navier–Stokes equations is as follows: search (u,p)∈X×M, such that

(14) $$\left(u_{t},v\right)+\nu a\left(u,v\right)-d\left(v,p\right)+d\left(u,q\right)+b\left(u,u,v\right)=\left(f,v\right),$$

(15) $$u\left(x,0\right)=u_{0}\left(x\right),$$

for all (v,q)∈X×M.

## 3. Long-Time Stability Analysis

Next, we will deduce that the solution of Equations (14) and (15) can be bounded by power-type constants with respect to the initial data, the viscosity, and the body force for all t>0.

### 3.1. Auxiliary Problem

To derive the stability of the unsteady Navier–Stokes equations, we firstly consider a linearized problem as follows:

(16) $${\hat{u}}_{t}-\nu \Delta \hat{u}+\left(\phi \cdot \nabla \right)\hat{u}+\nabla \hat{p}=f,\;\;\;\mathrm{div}\;\hat{u}=0\;\;\forall \left(x,t\right)\in \Omega \times \left(0,+\infty \right),$$

(17) $$\hat{u}\left(x,0\right)=u_{0}\left(x\right),\;\;\forall x\in \Omega ,\;\;\;\;\;\;\;\;\;\hat{u}{\left(x,t\right)|}_{\partial \Omega }=0,\;\;\forall t\in \left[0,+\infty \right),$$

where ϕ(x,t) is a given function that can be chosen as required in the following. Obviously, the variational formulation of (16) and (17) is as follows: search $(\hat{u},\hat{p})\in X\times M$, for t>0, such that

(18) $$\left({\hat{u}}_{t},v\right)+\nu a\left(\hat{u},v\right)-d\left(v,\hat{p}\right)+d\left(\hat{u},q\right)+b\left(\phi ,\hat{u},v\right)=\left(f,v\right),$$

(19) $$\hat{u}\left(x,0\right)=u_{0}\left(x\right).$$

for all (v,q)∈X×M.

**Lemma** **1.** *Assume that (* ***A1*** *) and (* ***A2*** *) hold, and $\left(\hat{u},\hat{p}\right)$ is the solution of problems (18)–(19). For t>0,*

 *(I).* *It is valid*
(20) $$|\hat{u}{|}^{2}+\frac{\nu }{2}e^{-\nu \lambda_{1}t}\int_{0}^{t}e^{\nu \lambda_{1}s}\left|\right|\hat{u}{\left|\right|}^{2}ds\leq e^{-\nu \lambda_{1}t}{\left|u_{0}\right|}^{2}+{\tilde{C}}_{f}^{2}:=\kappa_{1}^{2},$$
  *where ${\tilde{C}}_{f}^{2}=\frac{2C_{f}^{2}}{\nu^{2}\lambda_{1}}$.*
 *(II).* *If*
(21) $$\sigma_{1}:=\frac{N\kappa_{2}}{\nu }<1,$$
  *and we choose a function ϕ(x,t) that satisfies ∇·ϕ=0 and $\left||\phi |\right|\leq \kappa_{2}$, then it holds that*
(22) $$\left|\right|\hat{u}{\left|\right|}^{2}+\frac{1}{2}\nu e^{-\nu \lambda_{1}t}\int_{0}^{t}e^{\nu \lambda_{1}s}|A\hat{u}{|}^{2}ds\leq e^{-\nu \lambda_{1}t}\left|\right|u_{0}{\left|\right|}^{2}+{\tilde{C}}_{f}^{2}+5\kappa_{1}^{2}:=\kappa_{2}^{2},$$
(23) $$e^{-\nu \lambda_{1}t}\int_{0}^{t}e^{\nu \lambda_{1}s}\left(\right|{\hat{u}}_{s}{|}^{2}+\left|\right|\hat{p}{\left|\right|}_{1}^{2})ds\leq 2\left(\nu \kappa_{2}^{2}+{\tilde{C}}_{f}^{2}+\nu \kappa_{1}\kappa_{2}\right):=\kappa_{3}^{2}.$$
 *(III).* *Under the assumptions of (II) and choosing a function ϕ(x,t) satisfying $|\phi_{t}{|}^{2}+\nu e^{-\nu \lambda_{1}t}\int_{0}^{t}e^{\nu \lambda_{1}s}\left|\right|\phi_{s}{\left|\right|}^{2}ds\leq \kappa$, it holds that*
(24) $$|{\hat{u}}_{t}{|}^{2}+\nu e^{-\nu \lambda_{1}t}\int_{0}^{t}e^{\nu \lambda_{1}s}\left|\right|{\hat{u}}_{s}{\left|\right|}^{2}ds\leq \kappa ,$$
(25) $$\nu^{2}|A\hat{u}{|}^{2}+\left|\right|\hat{p}{\left|\right|}_{1}^{2}\leq \kappa .$$
*Hereafter, κ is a general power-type positive constant that may take different values at different occurrences.*

**Proof.**  Setting $\left(v,q\right)=e^{\nu \lambda_{1}t}\left(\hat{u},\hat{p}\right)$ in (18) and using (3) and (6), we have

(26) $$\frac{1}{2}\frac{d}{dt}e^{\nu \lambda_{1}t}|\hat{u}{|}^{2}+\frac{\nu }{2}e^{\nu \lambda_{1}t}\left|\right|\hat{u}{\left|\right|}^{2}\leq e^{\nu \lambda_{1}t}\left(f,\hat{u}\right).$$

Integrating (26) from 0 to *t*, using $|\left(f,\hat{u}\right)|\leq \frac{\nu }{4}\left|\right|\hat{u}{\left|\right|}^{2}+\frac{1}{\nu }{\left||f|\right|}_{-1}^{2}$, and multiplying by $e^{-\nu \lambda_{1}t}$, we obtain

$$|\hat{u}{|}^{2}+\frac{\nu }{2}e^{-\nu \lambda_{1}t}\int_{0}^{t}e^{\nu \lambda_{1}s}\left|\right|\hat{u}{\left|\right|}^{2}ds\leq e^{-\nu \lambda_{1}t}{\left|u_{0}\right|}^{2}+\frac{2C_{f}^{2}\left(1-e^{-\nu \lambda_{1}t}\right)}{\nu^{2}\lambda_{1}}\leq e^{-\nu \lambda_{1}t}{\left|u_{0}\right|}^{2}+\frac{2C_{f}^{2}}{\nu^{2}\lambda_{1}}.$$

On the other hand, applying *P* to (16), then taking the inner product with $v=e^{\nu \lambda_{1}t}A\hat{u}$ and using (4), we obtain

(27) $$\frac{1}{2}\frac{d}{dt}e^{\nu \lambda_{1}t}\left|\right|\hat{u}{\left|\right|}^{2}+\frac{\nu }{2}e^{\nu \lambda_{1}t}{\left|A\hat{u}\right|}^{2}+b\left(\phi ,\hat{u},e^{\nu \lambda_{1}t}A\hat{u}\right)=\left(f,e^{\nu \lambda_{1}t}A\hat{u}\right).$$

Thanks to

$$\begin{array}{cc} \;|(f,A\hat{u})| & \leq \frac{\nu }{4}\left|\right|\hat{u}{\left|\right|}^{2}+\frac{1}{\nu }{\left||f|\right|}_{1}^{2},\\ \;|b(\phi ,\hat{u},A\hat{u})| & \leq N||\phi ||\;||\hat{u}{\left|\right|}^{1/2}{\left|A\hat{u}\right|}^{3/2}\\ & \leq \frac{\nu }{4}|A\hat{u}{|}^{2}+\frac{N^{4}}{\nu^{3}}{\left||\phi |\right|}^{4}\left|\right|\hat{u}{\left|\right|}^{2}, \end{array}$$

it is valid that

$$\left|\right|\hat{u}{\left|\right|}^{2}+\frac{\nu }{2}e^{-\nu \lambda_{1}t}\int_{0}^{t}e^{\nu \lambda_{1}s}{\left|A\hat{u}\right|}^{2}ds\leq \kappa_{2}^{2},$$

by noting the assumption (21).

To derive (23), applying *P* to (16), we arrive at

$$|{\hat{u}}_{t}{|}^{2}\leq \nu^{2}|A\hat{u}{|}^{2}+{\left|f\right|}^{2}+N^{2}{\left||\phi |\right|}^{2}\left|\right|\hat{u}||\;|A\hat{u}|,$$

which, together with (21) and (22), implies

(28) $$e^{-\nu \lambda_{1}t}\int_{0}^{t}e^{\nu \lambda_{1}s}{\left|{\hat{u}}_{s}\right|}^{2}ds\leq \nu \kappa_{2}^{2}+{\tilde{C}}_{f}^{2}+\nu \kappa_{1}\kappa_{2}.$$

At the same time, (16) follows by

$$\left|\right|\hat{p}{\left|\right|}_{1}^{2}\leq |{\hat{u}}_{t}{|}^{2}+\nu^{2}|A\hat{u}{|}^{2}+{\left|f\right|}^{2}+N^{2}{\left||\phi |\right|}^{2}\left|\right|\hat{u}||\;|A\hat{u}|,$$

which, combining with (28), yields (23).

Differentiating (18) with respect to *t* yields

(29) $$\left({\hat{u}}_{tt},v\right)+\nu a\left({\hat{u}}_{t},v\right)-d\left(v,{\hat{p}}_{t}\right)+d\left({\hat{u}}_{t},q\right)+b\left(\phi_{t},\hat{u},v\right)+b\left(\phi ,{\hat{u}}_{t},v\right)=\left(f_{t},v\right).$$

Setting $\left(v,q\right)=e^{\nu \lambda_{1}t}\left({\hat{u}}_{t},{\hat{p}}_{t}\right)$ in (29) and using (6), we have

(30) $$\frac{1}{2}\frac{d}{dt}e^{\nu \lambda_{1}t}|{\hat{u}}_{t}{|}^{2}+\nu e^{\nu \lambda_{1}t}\left|\right|{\hat{u}}_{t}{\left|\right|}^{2}+b\left(\phi_{t},\hat{u},e^{\nu \lambda_{1}t}{\hat{u}}_{t}\right)=\left(f_{t},e^{\nu \lambda_{1}t}{\hat{u}}_{t}\right)+\frac{1}{2}\nu \lambda_{1}e^{\nu \lambda_{1}t}{\left|{\hat{u}}_{t}\right|}^{2}.$$

It is valid, by using (7), that

$$\begin{array}{cc} \;|(f_{t},{\hat{u}}_{t})| & \leq \frac{\nu }{4}\left|\right|{\hat{u}}_{t}{\left|\right|}^{2}+\frac{1}{\nu }\left|\right|f_{t}{\left|\right|}_{-1}^{2},\\ \;|b(\phi_{t},\hat{u},{\hat{u}}_{t})| & =|b(\phi_{t},{\hat{u}}_{t},\hat{u})|\\ & \leq \frac{\nu }{4}\left|\right|{\hat{u}}_{t}{\left|\right|}^{2}+\frac{N^{2}}{\nu }|\phi_{t}\left|\;|\right|\phi_{t}\left||\;|\right|\hat{u}{\left|\right|}^{2}. \end{array}$$

We have, after using (21)–(23), that

$$|{\hat{u}}_{t}{|}^{2}+\nu e^{-\nu \lambda_{1}t}\int_{0}^{t}e^{\nu \lambda_{1}s}\left|\right|{\hat{u}}_{t}{\left|\right|}^{2}ds\leq e^{-\nu \lambda_{1}t}{\left|{\hat{u}}_{t}\left(0\right)\right|}^{2}+\frac{2C_{f}^{2}}{\nu^{2}\lambda_{1}}+\nu \lambda_{1}\kappa_{3}^{2}+\sqrt{\nu }\kappa .$$

By (18), it holds that

$$|{\hat{u}}_{t}{\left(0\right)|}^{2}\leq \nu^{2}|Au_{0}{|}^{2}+{\left|f\left(0\right)\right|}^{2}+N|u_{0}|\;|Au_{0}\left|\;|\right|u_{0}{\left|\right|}^{2},$$

which, together with the above estimate, suggests (24).

Moreover, (18) yields

$$\begin{array}{cc} \;||\hat{p}{\left|\right|}_{1}^{2}+\nu^{2}{\left|A\hat{u}\right|}^{2} & \leq {\left|f\right|}^{2}+|{\hat{u}}_{t}{|}^{2}+N^{2}{\left||\phi |\right|}^{2}\left|\right|\hat{u}||\;|A\hat{u}|\\ & \leq \frac{1}{2}\nu^{2}|A\hat{u}{|}^{2}+{\left|f\right|}^{2}+|{\hat{u}}_{t}{|}^{2}+\frac{N^{4}}{2\nu^{2}}{\left||\phi |\right|}^{4}\left|\right|\hat{u}{\left|\right|}^{2}. \end{array}$$

Using (21), (22), and (24), we obtain (25). □

### 3.2. Long-Time Stability for the NSE

Now, we consider the following iterative scheme of problems (1) and (2): find $\left(u^{l},p^{l}\right)\in \left(X,M\right)\;\left(l=1,2,\cdots \right)$ such that

(31) $$u_{t}^{l}-\nu \Delta u^{l}+\left(u^{l-1}\cdot \nabla \right)u^{l}+\nabla p^{l}=f,\;\;\;\mathrm{div}\;u^{l}=0\;\;\forall \left(x,t\right)\in \Omega \times \left(0,+\infty \right),$$

(32) $$u^{l}\left(x,0\right)=u_{0}\left(x\right),\;\;\forall x\in \Omega ,\;\;\;\;\;\;\;\;\;u^{l}{\left(x,t\right)|}_{\partial \Omega }=0,\;\;\forall t\in \left[0,+\infty \right),$$

with $u^{0}\left(x,t\right)$ being a given initial guess that can be chosen as required. The weak formulation of (31) and (32) is as follows: find $\left(u^{l},p^{l}\right)\in \left(X,M\right)\;\left(l=1,2,\cdots \right)$ such that

(33) $$\left(u_{t}^{l},v\right)+\nu a\left(u^{l},v\right)-d\left(v,p^{l}\right)+d\left(u^{l},q\right)+b\left(u^{l-1},u^{l},v\right)=\left(f,v\right),$$

(34) $$u^{l}\left(x,0\right)=u_{0}\left(x\right),$$

for all (v,q)∈(X,M). Then, for the solutions $\left\{\left(u^{l},p^{l}\right)\right\}$ of (33) and (34), the following lemma holds.

**Lemma** **2.** *Assume that (* ***A1*** *) and (* ***A2*** *) hold, and $\left(u^{l},p^{l}\right)$ is the solution of problems (33)–(34). For all t>0,*

 *(I).* *It is valid that*
(35) $$|u^{l}{|}^{2}+\frac{1}{2}\nu e^{-\nu \lambda_{1}t}\int_{0}^{t}e^{\nu \lambda_{1}s}\left|\right|u^{l}{\left|\right|}^{2}ds\leq \kappa_{1}^{2}.$$
 *(II).* *Assuming that $\left(u_{0},\nu ,f\right)$ satisfies (21) and choosing an iterative initial guess $u^{0}\left(x,t\right)$ satisfying $divu^{0}\left(x,t\right)=0$ and $\left|\right|u^{0}\left(x,t\right)\left|\right|\leq \kappa_{2}$, it holds that*
(36) $$\left|\right|u^{l}{\left|\right|}^{2}+\frac{1}{2}\nu e^{-\nu \lambda_{1}t}\int_{0}^{t}e^{\nu \lambda_{1}s}{\left|Au^{l}\right|}^{2}ds\leq \kappa_{2}^{2},$$
(37) $$e^{-\nu \lambda_{1}t}\int_{0}^{t}e^{\nu \lambda_{1}s}\left(\right|u_{s}^{l}{|}^{2}+\left|\right|p^{l}{\left|\right|}_{1}^{2})ds\leq \kappa_{3}^{2}.$$
 *(III).* *Under the assumptions in II) and choosing an iterative initial guess $u^{0}\left(x,t\right)$ satisfying $|u_{t}^{0}{\left(x,t\right)|}^{2}+\nu e^{-\nu \lambda_{1}t}\int_{0}^{t}e^{\nu \lambda_{1}s}\left|\right|u_{s}^{0}\left(x,s\right){\left|\right|}^{2}ds\leq \kappa$, it holds that*
(38) $$|u_{t}^{l}{|}^{2}+\nu e^{-\nu \lambda_{1}t}\int_{0}^{t}e^{\nu \lambda_{1}s}\left|\right|u_{s}^{l}{\left|\right|}^{2}ds\leq \kappa ,$$
(39) $$\nu^{2}|Au^{l}{|}^{2}+\left|\right|p^{l}{\left|\right|}_{1}^{2}\leq \kappa .$$

**Proof.**  The proof is similar to Lemma 1 via the induction, which is omitted here. □

Next, by investigating the convergence of the sequence $\left(u^{l},p^{l}\right)$, we will prove that the solution of the unsteady Navier–Stokes equations is uniformly bounded by some power-type constants under some assumptions on $\left(\nu ,u_{0},f\right)$.

**Theorem** **1.** *Assume that (* ***A1*** *) and (* ***A2*** *) hold. For the solution (u,p) of the time-dependent Navier–Stokes Equations (14) and (15), it holds that*

(40) $${\left|u\right|}^{2}+\nu e^{-\nu \lambda_{1}t}\int_{0}^{t}e^{\nu \lambda_{1}s}{\left||u|\right|}^{2}ds\leq \kappa_{1}^{2}.$$

*Furthermore, assume that (21) holds; then, there exist subsequences $\left\{u^{l^{\prime }}\right\}$ in the solution sequence $\left\{u^{l}\right\}$ of problems (33)–(34) such that, as $l^{\prime }\to +\infty$,*

$$\begin{array}{cc} & u^{l^{\prime }}\to u\;weakly\;in\;L^{2,\nu }\left(0,+\infty ;X\right),\\ & u^{l^{\prime }}\to u\;weak-star\;in\;L^{\infty }\left(0,+\infty ;Y\right). \end{array}$$

**Proof.**  Inequality (40) can be proved by a similar process as that in Lemma 1, which is omitted here.

To prove the convergence results, let

$$w^{l}:=u^{l}-u^{l-1},\;\;\;\;\;\varsigma^{l}:=p^{l}-p^{l-1},\;\;\;\;\;l=1,2,\cdots .$$

From (33), it is easy to check that $\left(w^{l},\varsigma^{l}\right)\in \left(X,M\right)$ satisfies

(41) $$\left(w_{t}^{l},v\right)+\nu a\left(w^{l},v\right)-d\left(v,s^{l}\right)+d\left(w^{l},q\right)+b\left(u^{l-2},w^{l},v\right)+b\left(w^{l-1},u^{l},v\right)=0,$$

for all (v,q)∈(X,M).

Setting $\left(v,q\right)=e^{\nu \lambda_{1}t}\left(w^{l},\varsigma^{l}\right)$ in (41), we have

(42) $$\frac{1}{2}\frac{d}{dt}e^{\nu \lambda_{1}t}|w^{l}{|}^{2}-\frac{1}{2}\nu \lambda_{1}e^{\nu \lambda_{1}t}|w^{l}{|}^{2}+\nu e^{\nu \lambda_{1}t}\left|\right|w^{l}{\left|\right|}^{2}+b\left(w^{l-1},u^{l},e^{\nu \lambda_{1}t}w^{l}\right)=0.$$

Since

$$\begin{array}{cc} \;|b(w^{l-1},u^{l},w^{l})| & =|b\left(w^{l-1},w^{l},u^{l}\right)\left|\leq N\right|w^{l-1}\left|\;|\right|w^{l}||\;|Au^{l}|\\ & \leq \frac{\nu }{2}\left|\right|w^{l}{\left|\right|}^{2}+\frac{N^{2}}{2\nu }|Au^{l}{|}^{2}{\left|w^{l-1}\right|}^{2},\\ \frac{1}{2}\nu \lambda_{1}{\left|w^{l}\right|}^{2} & \leq \frac{1}{2}\nu ||w^{l}{\left|\right|}^{2}, \end{array}$$

and noting $w^{l}\left(x,0\right)=u^{l}\left(x,0\right)-u^{l-1}\left(x,0\right)=u_{0}\left(x\right)-u_{0}\left(x\right)=0$, we obtain

$$\begin{array}{cc} \;|w^{l}{|}^{2} & \leq \frac{N^{2}}{\nu^{2}}\left(\nu e^{-\nu \lambda_{1}t}\int_{0}^{t}e^{\nu \lambda_{1}s}{\left|Au^{l}\right|}^{2}ds\right){\left|w^{l-1}\right|}^{2}\\ & \leq \frac{N^{2}\kappa_{2}^{2}}{\nu^{2}}|w^{l-1}{|}^{2}=\sigma_{1}^{2}|w^{l-1}{|}^{2}\leq \sigma_{1}^{2l}{\left|w^{0}\right|}^{2}. \end{array}$$

Letting l→+∞ in the above inequality and using (21), we obtain

(43) $$\lim_{l\to +\infty }{\left|w^{l}\right|}^{2}=0.$$

On the other hand, it holds that

$$\begin{array}{cc} \;|b(w^{l-1},u^{l},w^{l})| & =|b\left(w^{l-1},w^{l},u^{l}\right)\left|\leq N|\right|w^{l-1}\left||\;|\right|w^{l}\left||\;|\right|u^{l}\left|\right|\\ & \leq \frac{\nu }{2}\left|\right|w^{l}{\left|\right|}^{2}+\frac{N^{2}}{2\nu }\left|\right|u^{l}{\left|\right|}^{2}\left|\right|w^{l-1}{\left|\right|}^{2}. \end{array}$$

Equation (42) yields

$$\begin{array}{cc} \;e^{-\nu \lambda_{1}t}\int_{0}^{t}e^{\nu \lambda_{1}s}\left|\right|w^{l}{\left|\right|}^{2}ds & \leq \frac{N^{2}}{\nu^{2}}\left|\right|u^{l}{\left|\right|}^{2}e^{-\nu \lambda_{1}t}\int_{0}^{t}e^{\nu \lambda_{1}s}\left|\right|w^{l-1}{\left|\right|}^{2}ds+\lambda_{1}e^{-\nu \lambda_{1}t}\int_{0}^{t}e^{\nu \lambda_{1}s}ds{\left|w^{l}\right|}^{2}\\ & \leq \sigma_{1}^{2l}\left(e^{-\nu \lambda_{1}t}\int_{0}^{t}e^{\nu \lambda_{1}s}\left|\right|w^{0}{\left|\right|}^{2}ds\right)+\frac{\left(1-\sigma_{1}^{2l}\right)\left(1-e^{-\nu \lambda_{1}t}\right)}{\nu (1-\sigma_{1}^{2})}{\left|w^{l}\right|}^{2}, \end{array}$$

which suggests, by using (43), that

(44) $$\lim_{l\to +\infty }e^{-\nu \lambda_{1}t}\int_{0}^{t}e^{\nu \lambda_{1}s}\left|\right|w^{l}{\left|\right|}^{2}ds=0.$$

Thus, there exist Cauchy subsequences $\left\{u^{l^{\prime }}\right\}$ in both $L^{\infty }\left(0,+\infty ;Y\right)$ and $L^{2,\nu }\left(0,+\infty ;X\right)$, such that $u:=\lim_{l^{\prime }\to +\infty }u^{l^{\prime }}$ is the solution of the time-dependent Navier–Stokes Equations (14) and (15). The proof is completed. □

**Theorem** **2.** *Under the assumptions of Theorem 1, there exist subsequences $\left\{u^{l^{\prime }}\right\},\left\{u_{t}^{l^{\prime }}\right\}$, and $\left\{p^{l^{\prime }}\right\}$ in the solution sequences $\left\{u^{l}\right\},\left\{u_{t}^{l}\right\}$, and $\left\{p^{l}\right\}$ of problems (33) and (34) such that, as $l^{\prime }\to +\infty$,*

$$\begin{array}{c} \;u^{l^{\prime }}\to u\;weak-star\;in\;L^{\infty }\left(0,+\infty ;X\right),\;\\ \;u^{l^{\prime }}\to u\;weakly\;in\;L^{2,\nu }\left(0,+\infty ;D\left(A\right)\right),\\ \;u_{t}^{l^{\prime }}\to u_{t}\;weakly\;in\;L^{2,\nu }\left(0,+\infty ;Y\right),\\ p^{l^{\prime }}\to p\;weakly\;in\;L^{2,\nu }\left(0,+\infty ;M\cap H^{1}\left(\Omega \right)\right), \end{array}$$

*and*

(45) $${\left||u|\right|}^{2}+\nu e^{-\nu \lambda_{1}t}\int_{0}^{t}e^{\nu \lambda_{1}s}{\left|Au\right|}^{2}ds\leq \kappa_{2}^{2},$$

(46) $$e^{-\nu \lambda_{1}t}\int_{0}^{t}e^{\nu \lambda_{1}s}\left(\right|u_{s}{|}^{2}+{\left||p|\right|}_{1}^{2})ds\leq \kappa_{3}^{2}.$$

**Proof.**  Differentiating $d(w^{l},q)$ with respect to *t* in (41) and setting $\left(v,q\right)=e^{\nu \lambda_{1}t}\left(w_{t}^{l},\varsigma^{l}\right)$, we obtain

$$e^{\nu \lambda_{1}t}|w_{t}^{l}{|}^{2}+\frac{\nu }{2}\frac{d}{dt}e^{\nu \lambda_{1}t}\left|\right|w^{l}{\left|\right|}^{2}+b\left(u^{l-2},w^{l},e^{\nu \lambda_{1}t}w_{t}^{l}\right)+b\left(w^{l-1},u^{l},e^{\nu \lambda_{1}t}w_{t}^{l}\right)=\frac{\nu }{2}\nu \lambda_{1}e^{\nu \lambda_{1}t}\left|\right|w^{l}{\left|\right|}^{2}.$$

Due to

$$\begin{array}{cc} \;|b(u^{l-2},w^{l},w_{t}^{l})| & \leq N|u^{l-2}{|}^{1/2}|Au^{l-2}{|}^{1/2}\left|\right|w^{l}\left||\;\right|w_{t}^{l}|\\ & \leq \frac{1}{4}|w_{t}^{l}{|}^{2}+N^{2}|u^{l-2}|\;|Au^{l-2}\left|\;|\right|w^{l}{\left|\right|}^{2},\\ \;|b(w^{l-1},u^{l},w_{t}^{l})| & \leq N||w^{l-1}\left||\;|\right|u^{l}{\left|\right|}^{1/2}|Au^{l}{|}^{1/2}\;\left|w_{t}^{l}\right|\\ & \leq \frac{1}{4}|w_{t}^{l}{|}^{2}+N^{2}\left|\right|u^{l}||\;|Au^{l}\left|\;|\right|w^{l-1}{\left|\right|}^{2}, \end{array}$$

we obtain

$$\begin{array}{cc} \;\nu ||w^{l}{\left|\right|}^{2}+e^{-\nu \lambda_{1}t}\int_{0}^{t}e^{\nu \lambda_{1}s}{\left|w_{s}^{l}\right|}^{2}ds\leq & \frac{\nu }{2}\nu \lambda_{1}e^{-\nu \lambda_{1}t}\int_{0}^{t}e^{\nu \lambda_{1}s}\left|\right|w^{l}{\left|\right|}^{2}ds\\ & +N^{2}|u^{l-2}|\;|Au^{l-2}|e^{-\nu \lambda_{1}t}\int_{0}^{t}e^{\nu \lambda_{1}s}\left|\right|w^{l}{\left|\right|}^{2}ds\\ & +N^{2}\left|\right|u^{l}||\;|Au^{l}|e^{-\nu \lambda_{1}t}\int_{0}^{t}e^{\nu \lambda_{1}s}\left|\right|w^{l-1}{\left|\right|}^{2}ds. \end{array}$$

Letting l→+∞ in the above inequality and using Lemma 2 and (44), it holds that

(47) $$\lim_{l\to +\infty }\left|\right|w^{l}{\left|\right|}^{2}+\lim_{l\to +\infty }e^{-\nu \lambda_{1}t}\int_{0}^{t}e^{\nu \lambda_{1}s}{\left|w_{s}^{l}\right|}^{2}ds=0.$$

Hence, there exist Cauchy subsequences $\left\{u^{l^{\prime }}\right\}$ in $L^{\infty }\left(0,+\infty ;X\right)$ and $\left\{u_{t}^{l^{\prime }}\right\}$ in $L^{2,\nu }\left(0,+\infty ;Y\right)$ such that

$${\left||u|\right|}^{2}:=\lim_{l^{\prime }\to +\infty }\left|\right|u^{l^{\prime }}{\left|\right|}^{2}\leq \kappa_{2}^{2},$$

and

$$e^{-\nu \lambda_{1}t}\int_{0}^{t}e^{\nu \lambda_{1}s}|u_{s}{|}^{2}ds:=\lim_{l^{\prime }\to +\infty }e^{-\nu \lambda_{1}t}\int_{0}^{t}e^{\nu \lambda_{1}s}{\left|u_{s}^{l^{\prime }}\right|}^{2}ds\leq \kappa_{3}^{2}.$$

From (41), we have

$$\begin{array}{cc} & \nu^{2}|Aw^{l}{|}^{2}+\left|\right|\varsigma^{l}{\left|\right|}_{1}^{2}\\ \;\leq & |w_{t}^{l}{|}^{2}+N^{2}|u^{l-2}\left|\;|\right|u^{l-2}\left||\;|\right|w^{l}||\;|Aw^{l}|+N^{2}|w^{l-1}\left|\;|\right|w^{l-1}\left||\;|\right|u^{l}||\;|Au^{l}|\\ \leq & \frac{\nu^{2}}{2}|Aw^{l}{|}^{2}+\frac{N^{4}}{2\nu^{2}}|u^{l-2}{|}^{2}\left|\right|u^{l-2}{\left|\right|}^{2}\left|\right|w^{l}{\left|\right|}^{2}+|w_{t}^{l}{|}^{2}+N^{2}|w^{l-1}\left|\;|\right|w^{l-1}\left||\;|\right|u^{l}||\;|Au^{l}|. \end{array}$$

Integrating above inequalities from 0 to *t*, letting l→+∞, and using Lemma 2, (43), (44), and (47), we arrive at

(48) $$\lim_{l\to +\infty }e^{-\nu \lambda_{1}t}\int_{0}^{t}e^{\nu \lambda_{1}s}|Aw^{l}{|}^{2}ds+\lim_{l\to +\infty }e^{-\nu \lambda_{1}t}\int_{0}^{t}e^{\nu \lambda_{1}s}\left|\right|\varsigma^{l}{\left|\right|}_{1}^{2}ds=0.$$

Hence, there exist convergent Cauchy subsequences $\left\{Au^{l^{\prime }}\right\}$ in $L^{2,\nu }\left(0,+\infty ;Y\right)$ and $\left\{p_{t}^{l^{\prime }}\right\}$ in $L^{2,\nu }\left(0,+\infty ;M\cap H^{1}\left(\Omega \right)\right)$ such that

$$e^{-\nu \lambda_{1}t}\int_{0}^{t}e^{\nu \lambda_{1}s}{\left|Au\right|}^{2}ds:=\lim_{l^{\prime }\to +\infty }e^{-\nu \lambda_{1}t}\int_{0}^{t}e^{\nu \lambda_{1}s}{\left|Au^{l^{\prime }}\right|}^{2}ds\leq \kappa_{2}^{2},$$

and

$$e^{-\nu \lambda_{1}t}\int_{0}^{t}e^{\nu \lambda_{1}s}{\left||p|\right|}_{1}^{2}ds:=\lim_{l^{\prime }\to +\infty }e^{-\nu \lambda_{1}t}\int_{0}^{t}e^{\nu \lambda_{1}s}\left|\right|p^{l^{\prime }}{\left|\right|}_{1}^{2}ds\leq \kappa_{3}^{2}.$$

The proof is completed. □

**Theorem** **3.** *Under the assumptions of Theorem 1, it holds that*

(49) $$|u_{t}{|}^{2}+\nu e^{-\nu \lambda_{1}t}\int_{0}^{t}e^{\nu \lambda_{1}s}\left|\right|u_{s}{\left|\right|}^{2}ds\leq e^{-\nu \lambda_{1}t}{\tilde{C}}_{0}^{2}+\left(\nu +\nu \lambda_{1}+1\right)\kappa_{3}^{2}+\nu {\tilde{C}}_{f}^{2}:=\kappa_{4}^{2},$$

(50) $$\nu^{2}{\left|Au\right|}^{2}+{\left||p|\right|}_{1}^{2}\leq \nu^{2}\kappa_{1}^{2}+2\kappa_{4}^{2}+2C_{f}^{2},$$

(51) $$\tau \left(t\right)||u_{t}{\left|\right|}^{2}+\nu e^{-\nu \lambda_{1}t}\int_{0}^{t}e^{\nu \lambda_{1}s}\tau \left(s\right){\left|Au_{s}\right|}^{2}ds\leq 2\left(\frac{1+\nu \lambda_{1}}{\nu }+5\right)\kappa_{4}^{2}+2{\tilde{C}}_{f}^{2},$$

(52) $$e^{-\nu \lambda_{1}t}\int_{0}^{t}e^{\nu \lambda_{1}s}\tau \left(s\right)(|u_{ss}{|}^{2}+\left|\right|p_{s}{\left|\right|}_{1}^{2})ds\leq \left[1+\left(\lambda_{1}+9\right)\nu +\sqrt{\nu (1+\nu \lambda_{1})}\right]\kappa_{4}^{2}+\nu {\tilde{C}}_{f}^{2},$$

*where ${\tilde{C}}_{0}^{2}=C_{f}^{2}+\nu^{2}C_{0}^{2}+N^{2}C_{0}^{4}$.*

**Proof.**  Differentiating (14) with respect to *t* yields

(53) $$\left(u_{tt},v\right)+\nu a\left(u_{t},v\right)-d\left(v,p_{t}\right)+d\left(u_{t},q\right)+b\left(u_{t},u,v\right)+b\left(u,u_{t},v\right)=\left(f_{t},v\right).$$

Taking $\left(v,q\right)=e^{\nu \lambda_{1}t}\left(u_{t},p_{t}\right)$ in (53) and using (6), we have

(54) $$\frac{1}{2}\frac{d}{dt}e^{\nu \lambda_{1}t}|u_{t}{|}^{2}+\nu e^{\nu \lambda_{1}t}\left|\right|u_{t}{\left|\right|}^{2}+b\left(u_{t},u,e^{\nu \lambda_{1}t}u_{t}\right)=\frac{1}{2}\nu \lambda_{1}e^{\nu \lambda_{1}t}{\left|u_{t}\right|}^{2}+\left(f_{t},e^{\nu \lambda_{1}t}u_{t}\right).$$

Since (7) follows

$$|b\left(u_{t},u,u_{t}\right)\left|\leq N\right|u_{t}\left|\;|\right|u_{t}\left||\;||u|\right|\leq \frac{\nu }{2}\left|\right|u_{t}{\left|\right|}^{2}+\frac{N^{2}}{2\nu }|u_{t}{|}^{2}{\left||u|\right|}^{2},$$

integrating (54) from 0 to *t*, multiplying by $e^{-\nu \lambda_{1}t}$, and noting

$$|u_{t}{\left(0\right)|}^{2}\leq {\left|f\left(0\right)\right|}^{2}+\nu^{2}|Au_{0}{|}^{2}+N^{2}\left|\right|u_{0}{\left|\right|}^{2}{\left|Au_{0}\right|}^{2}:={\tilde{C}}_{0}^{2},$$

we obtain

$$|u_{t}{|}^{2}+\nu e^{-\nu \lambda_{1}t}\int_{0}^{t}e^{\nu \lambda_{1}s}\left|\right|u_{s}{\left|\right|}^{2}ds=e^{-\nu \lambda_{1}t}{\tilde{C}}_{0}^{2}+\left(\nu 1+\nu \lambda_{1}+1\right)\kappa_{3}^{2}+\nu {\tilde{C}}_{f}^{2},$$

which implies (49).

On the other hand, (14) yields

$$\begin{array}{cc} \;\nu^{2}{\left|Au\right|}^{2}+{\left||p|\right|}_{1}^{2}\leq & |u_{t}{|}^{2}+N^{2}{\left|u|\;||u|\right|}^{2}\left|Au\right|+{\left|f\right|}^{2}\\ \;\leq & \frac{\nu^{2}}{2}{\left|Au\right|}^{2}+\frac{N^{4}}{2\nu^{2}}{\left|u\right|}^{2}{\left||u|\right|}^{4}+|u_{t}{|}^{2}+{\left|f\right|}^{2}\\ \leq & \frac{\nu^{2}}{2}{\left|Au\right|}^{2}+\frac{\nu^{2}\kappa_{1}^{2}}{2}+\kappa_{4}^{2}+{\left|f\right|}^{2}. \end{array}$$

We obtain (50).

Applying *P* to (53) and taking $v=e^{\nu \lambda_{1}t}\tau \left(t\right)Au_{t}$, we arrive at

(55) $$\begin{array}{cc} & \frac{1}{2}\frac{d}{dt}e^{\nu \lambda_{1}t}\tau \left(t\right)||u_{t}{\left|\right|}^{2}+\nu e^{\nu \lambda_{1}t}\tau \left(t\right){\left|Au_{t}\right|}^{2}+b\left(u_{t},u,e^{\nu \lambda_{1}t}\tau \left(t\right)Au_{t}\right)+b\left(u,u_{t},e^{\nu \lambda_{1}t}\tau \left(t\right)Au_{t}\right)\\ = & \frac{1}{2}e^{\nu \lambda_{1}t}\left|\right|u_{t}{\left|\right|}^{2}+\frac{1}{2}\nu \lambda_{1}e^{\nu \lambda_{1}t}\tau \left(t\right)||u_{t}{\left|\right|}^{2}+\left(f_{t},e^{\nu \lambda_{1}t}\tau \left(t\right)Au_{t}\right). \end{array}$$

By (11) and (12), it holds that

$$\begin{array}{cc} \;|(f_{t},e^{\nu \lambda_{1}t}\tau \left(t\right)Au_{t})|\leq & \frac{\nu \tau (t)}{4}|Au_{t}{|}^{2}+\frac{1}{\nu }{\left|f_{t}\right|}^{2},\\ \;|b(u_{t},u,\tau \left(t\right)Au_{t})|\leq & \frac{\nu \tau (t)}{4}|Au_{t}{|}^{2}+\frac{N^{2}\tau \left(t\right)}{\nu }|u_{t}\left|\;|\right|u_{t}||\;||u||\;|Au|,\\ |b(u,u_{t},\tau \left(t\right)Au_{t})|\leq & \frac{\nu \tau (t)}{4}|Au_{t}{|}^{2}+\frac{4N^{4}\tau \left(t\right)}{\nu^{3}}{\left||u|\right|}^{4}\left|\right|u_{t}{\left|\right|}^{2}. \end{array}$$

Substituting these inequalities into (55), integrating from 0 to *t*, multiplying by $e^{-\nu \lambda_{1}t}$, and using Theorems 1 and 2 and (49), we obtain

$$\begin{array}{cc} & \tau \left(t\right)||u_{t}{\left|\right|}^{2}+\frac{\nu }{2}e^{-\nu \lambda_{1}t}\int_{0}^{t}e^{\nu \lambda_{1}t}\tau \left(s\right){\left|Au_{s}\right|}^{2}ds\\ \leq & \frac{1+\nu \lambda_{1}}{\nu }\kappa_{4}^{2}+{\tilde{C}}_{f}^{2}+\frac{N^{2}}{\nu }|u_{t}\left|\;||u|\right|e^{-\nu \lambda_{1}t}\int_{0}^{t}e^{\nu \lambda_{1}s}\left|\right|u_{s}||\;|Au|ds\\ & +\frac{4N^{4}}{\nu^{3}}{\left||u|\right|}^{4}e^{-\nu \lambda_{1}t}\int_{0}^{t}e^{\nu \lambda_{1}s}\left|\right|u_{s}{\left|\right|}^{2}ds\\ \leq & \frac{1+\nu \lambda_{1}}{\nu }\kappa_{4}^{2}+5\kappa_{4}^{2}+{\tilde{C}}_{f}^{2}. \end{array}$$

Finally, it follows from (53) that

$$\begin{array}{c} |u_{tt}{|}^{2}+\left|\right|p_{t}{\left|\right|}_{1}^{2}\leq \nu^{2}|Au_{t}{|}^{2}+N^{2}|u_{t}\left|\;|\right|u_{t}\left||\;||u||\;|Au\right|+N^{2}\left|u|\;||u||\;|\right|u_{t}||\;|Au_{t}\left|+\right|f_{t}{|}^{2}. \end{array}$$

Thus, by using Theorems 1 and 2, and (49)–(51), we obtain

$$\begin{array}{cc} & e^{-\nu \lambda_{1}t}\int_{0}^{t}e^{\nu \lambda_{1}s}\tau \left(s\right)(|u_{ss}{|}^{2}+\left|\right|p_{s}{\left|\right|}_{1}^{2})ds\\ \leq & e^{-\nu \lambda_{1}t}\int_{0}^{t}e^{\nu \lambda_{1}s}\tau \left(s\right)(\nu^{2}|Au_{s}{|}^{2}+N^{2}|u_{s}\left|\;|\right|u_{s}\left||\;||u||\;|Au\right|+N^{2}{\left||u|\right|}^{2}\left|\right|u_{s}||\;|Au_{s}\left|\right)ds+\nu {\tilde{C}}_{f}^{2}\\ \leq & \nu \left(\frac{1+\nu \lambda_{1}}{\nu }+5\right)\kappa_{4}^{2}+\nu \kappa_{4}^{2}+\nu \sqrt{\frac{1+\nu \lambda_{1}}{\nu }+5}\kappa_{4}^{2}+\nu {\tilde{C}}_{f}^{2}. \end{array}$$

□

## 4. Long-Time Error Estimate

Let 0<h<1, and $\left(X_{h},M_{h}\right)\subset \left(X,M\right)$ be finite-dimensional subspaces for the velocity and pressure, which are characterized by $\tau_{h}$ with the mesh size *h* and assumed to be uniformly regular in the usual sense. We refer the reader to [28] for more details. We define the $L^{2}$-orthogonal projection operator $P_{h}:Y\to X_{h}$ by

$$\begin{array}{c} \left(P_{h}v,v_{h}\right)=\left(v,v_{h}\right)\;\;\;\forall v\in Y,v_{h}\in X_{h}, \end{array}$$

which follows by the properties (see [1,26,29])

(56) $$|v-P_{h}v|+h||v-P_{h}v||\leq ch||v||\;\;\;\forall v\in X,$$

(57) $$|v-P_{h}v|+h||v-P_{h}v||\leq ch^{2}\left|Av\right|\;\;\;\forall v\in D\left(A\right).$$

We also introduce the discrete analogues $V_{h}=\left\{v_{h}\in X_{h}|\mathrm{div}\;v_{h}=0\right\}$ and $A_{h}=-P_{h}\Delta_{h}$ of the Stokes operator *A* as

$$\begin{array}{c} \left(-\Delta_{h}u_{h},v_{h}\right)=\left(A_{h}^{1/2}u_{h},A_{h}^{1/2}v_{h}\right)=\left(\left(u_{h},v_{h}\right)\right)\;\;\;\;\forall u_{h},v_{h}\in X_{h}. \end{array}$$

Furthermore, we assume that the above finite element spaces $\left(X_{h},M_{h}\right)$ satisfy the following properties (see [26,27,29,30]):

**(A3)**. For each $v\in H^{2}{\left(\Omega \right)}^{2}\cap V$ and $q\in H^{1}\left(\Omega \right)\cap M$, there exist approximations $\pi_{h}v\in V_{h}$ and $\rho_{h}q\in M_{h}$ such that

(58) $$|v-\pi_{h}v|+h||v-\pi_{h}v||\leq ch^{k}{\left||v|\right|}_{k},\;\;\;k=1,2,$$

(59) $$|q-\rho_{h}q|\leq ch^{k}{\left||q|\right|}_{k},\;\;\;k=0,1,$$

together with the inverse inequality

$$\begin{array}{c} \left|\right|v_{h}\left|\right|\leq ch^{-1}\left|v_{h}\right|,\;\;\;v_{h}\in X_{h}, \end{array}$$

and the so-called inf-sup inequality: for each $q_{h}\in M_{h}$ such that

(60) $$\beta_{h}\left|q_{h}\right|\leq \underset{v_{h}\in X,v_{h}\neq 0}{\sup}\frac{d(v_{h},q_{h})}{\left|\right|v_{h}\left|\right|},$$

where $\beta_{h}$ is a positive constant depending on Ω.

For examples of element pairs satisfying the assumption (**A3**), we refer to the P2−P0 finite element pairs and the P1b−P1 mini finite element pairs (see [30,31]).

With the above notations, the finite element variational formulation for (33) and (34) and (14) and (15) are, respectively, as follows: find $\left(u_{h}^{l},p_{h}^{l}\right)\in \left(X_{h},M_{h}\right)\;\left(l=1,2,\cdots \right)$ such that

(61) $$\left(u_{ht}^{l},v_{h}\right)+\nu a\left(u_{h}^{l},v_{h}\right)-d\left(v_{h},p_{h}^{l}\right)+d\left(u_{h}^{l},q_{h}\right)+b\left(u_{h}^{l-1},u_{h}^{l},v_{h}\right)=\left(f,v_{h}\right),$$

(62) $$u_{h}^{l}\left(0\right)=u_{0h}^{l}=P_{h}u_{0},$$

and find $\left(u_{h},p_{h}\right)\in X_{h}\times M_{h}$ such that

(63) $$\left(u_{ht},v_{h}\right)+\nu a\left(u_{h},v_{h}\right)-d\left(v_{h},p_{h}\right)+d\left(u_{h},q_{h}\right)+b\left(u_{h},u_{h},v_{h}\right)=\left(f,v_{h}\right),$$

(64) $$u_{h}\left(0\right)=u_{0h}=P_{h}u_{0},$$

for all $\left(v_{h},q_{h}\right)\in X_{h}\times M_{h}$.

To derive power-type error estimates for the finite element solution, we need the Galerkin projection $\left(R_{h},Q_{h}\right)=\left(R_{h}\left(u,p\right),Q_{h}\left(u,p\right)\right):\left(X,M\right)\to \left(X_{h},M_{h}\right)$, which is defined in [1,30]

(65) $$\begin{array}{cc} & \nu a\left(u-R_{h},v_{h}\right)-d\left(v_{h},p-Q_{h}\right)+d\left(u-R_{h},q_{h}\right)=0,\\ & \;\;\;\;\;\;\;\;\forall \left(u,p\right)\in \left(X,M\right),\left(v_{h},q_{h}\right)\in \left(X_{h},M_{h}\right). \end{array}$$

**Lemma** **3.** *The Galerkin projection $\left(R_{h},Q_{h}\right)=\left(R_{h}\left(u,p\right),Q_{h}\left(u,p\right)\right)$, defined in (65), satisfies $\forall \left(u,p\right)\in (H^{2}{\left(\Omega \right)}^{2}\cap V,H^{1}\left(\Omega \right)\cap M)$, such that*

(66) $$\begin{array}{cc} & \nu |u-R_{h}\left(u,p\right)|+h(\nu ||u-R_{h}\left(u,p\right)||+|p-Q_{h}\left(u,p\right)\left|\right)\\ \leq & ch^{k}{\left(\nu ||u|\right|}_{k}+{\left||p|\right|}_{k-1}), \end{array}$$

(67) $$\begin{array}{cc} & \nu |u_{t}-R_{h}\left(u_{t},p_{t}\right)\left|+h(\nu |\right|u_{t}-R_{h}\left(u_{t},p_{t}\right)\left||+\right|p_{t}-Q_{h}\left(u_{t},p_{t}\right)\left|\right)\\ \leq & ch^{k}\left(\nu |\right|u_{t}{\left|\right|}_{k}+\left|\right|p_{t}{\left|\right|}_{k-1}), \end{array}$$

*with k=1,2.*

**Proof.**  The proof is very similar to that in proving Lemma 1 in [1,30], which is omitted here. □

### 4.1. Stability for Finite Element Solution

Similar to the continuous problems in Section 3, for the finite element variational formulations of (61)–(64), we can derive the following lemma and theorem.

**Lemma** **4.** *Under the assumptions of Lemma 2 and (* ***A3*** *), for the solution $\left(u_{h}^{l},p_{h}^{l}\right)$ of problems (61)–(62), it holds that*

(68) $$\begin{array}{cc} |u_{h}^{l}{|}^{2}+\nu e^{-\nu \lambda_{1}t}\int_{0}^{t}e^{\nu \lambda_{1}s}\left|\right|u_{h}^{l}{\left|\right|}^{2}ds & \leq \kappa_{1}^{2}, \end{array}$$

(69) $$\begin{array}{cc} \left|\right|u_{h}^{l}{\left|\right|}^{2}+\nu e^{-\nu \lambda_{1}t}\int_{0}^{t}e^{\nu \lambda_{1}s}{\left|A_{h}u_{h}^{l}\right|}^{2}ds & \leq \kappa_{2}^{2}, \end{array}$$

(70) $$\begin{array}{cc} e^{-\nu \lambda_{1}t}\int_{0}^{t}e^{\nu \lambda_{1}s}\left(\right|u_{hs}^{l}{|}^{2}+\left|\right|p_{h}^{l}{\left|\right|}_{1}^{2})ds & \leq \kappa_{3}^{2}. \end{array}$$

**Theorem** **4.** *Under the assumptions of Theorem 1 and (* ***A3*** *), for the finite element solution of the time-dependent Navier–Stokes Equations (63) and (64), it holds that*

(71) $$|u_{h}{|}^{2}+\nu e^{-\nu \lambda_{1}t}\int_{0}^{t}e^{\nu \lambda_{1}s}\left|\right|u_{h}{\left|\right|}^{2}ds\leq \kappa_{1}^{2},$$

(72) $$\left|\right|u_{h}{\left|\right|}^{2}+\nu e^{-\nu \lambda_{1}t}\int_{0}^{t}e^{\nu \lambda_{1}s}{\left|A_{h}u_{h}\right|}^{2}ds\leq \kappa_{2}^{2},$$

(73) $$e^{-\nu \lambda_{1}t}\int_{0}^{t}e^{\nu \lambda_{1}s}\left(\right|u_{hs}{|}^{2}+\left|\right|p_{h}{\left|\right|}_{1}^{2})ds\leq \kappa_{3}^{2},$$

(74) $$|u_{ht}{|}^{2}+\nu e^{-\nu \lambda_{1}t}\int_{0}^{t}e^{\nu \lambda_{1}s}\left|\right|u_{hs}{\left|\right|}^{2}ds\leq \kappa_{4}^{2},$$

(75) $$\nu^{2}|A_{h}u_{h}{|}^{2}+\left|\right|p_{h}{\left|\right|}_{1}^{2}\leq \nu^{2}\kappa_{1}^{2}+2\kappa_{4}^{2}+2C_{f}^{2},$$

(76) $$\tau \left(t\right)||u_{ht}{\left|\right|}^{2}+\nu e^{-\nu \lambda_{1}t}\int_{0}^{t}e^{\nu \lambda_{1}s}\tau \left(s\right){\left|A_{h}u_{hs}\right|}^{2}ds\leq 2\left(\frac{1+\nu \lambda_{1}}{\nu }+5\right)\kappa_{4}^{2}+2{\tilde{C}}_{f}^{2},$$

(77) $$e^{-\nu \lambda_{1}t}\int_{0}^{t}e^{\nu \lambda_{1}s}\tau \left(s\right)(|u_{hss}{|}^{2}+\left|\right|p_{hs}{\left|\right|}_{1}^{2})ds\leq \left[1+\left(\lambda_{1}+9\right)\nu +\sqrt{\nu (1+\nu \lambda_{1})}\right]\kappa_{4}^{2}+\nu {\tilde{C}}_{f}^{2}.$$

*Furthermore, there exist subsequences $\left\{u_{h}^{l^{\prime }}\right\}$ in the solution sequence $\left\{u_{h}^{l}\right\}$ of problems (61)–(62) such that, as $l^{\prime }\to +\infty$,*

$$\begin{array}{cc} & u_{h}^{l^{\prime }}\to u_{h}\;weakly\;in\;L^{2,\nu }\left(0,+\infty ;X\right),\\ & u_{h}^{l^{\prime }}\to u_{h}\;weak-star\;in\;L^{\infty }\left(0,+\infty ;Y\right),\\ & u_{ht}^{l^{\prime }}\to u_{ht}\;weakly\;in\;L^{2,\nu }\left(0,T;Y\right),\\ & p_{h}^{l^{\prime }}\to p_{h}\;weakly\;in\;L^{2,\nu }\left(0,T;M\cap H^{1}\left(\Omega \right)\right). \end{array}$$

### 4.2. Error Estimate

**Lemma** **5.** *Under the assumptions of Theorem 4, let (u,p) and $\left(u_{h},p_{h}\right)$ be the solutions of problems (14) and (15), and (63) and (64), respectively. If*

(78) $$\begin{array}{c} \sigma_{2}:=\frac{N\kappa_{2}}{\nu \sqrt{1-\epsilon }}<1,\;\;\;\;\forall \epsilon \in \left(0,1\right), \end{array}$$

*it holds that*

(79) $$\begin{array}{c} |u-u_{h}{|}^{2}\leq \frac{\kappa_{5}^{2}}{1-\sigma_{2}^{2}}h^{2}, \end{array}$$

(80) $$\begin{array}{c} e^{-\nu \lambda_{1}t}\int_{0}^{t}e^{\nu \lambda_{1}s}||u-u_{h}{\left|\right|}^{2}ds\leq \kappa_{6}^{2}h^{2}, \end{array}$$

*where $\kappa_{5}^{2}$ and $\kappa_{6}^{2}$ are power-type functions satisfying $\kappa_{5}^{2}:=c[||u_{0}{\left|\right|}^{2}+\left(\nu \kappa_{1}^{2}+\kappa_{3}^{2}\right)+\kappa_{3}\left(\kappa_{2}\nu^{-1/2}+\kappa_{3}\nu^{-1}\right)$$+\frac{\sigma_{2}^{2}}{\epsilon }\left(\kappa_{2}^{2}+\kappa_{3}^{2}\nu^{-1}\right)+\frac{\sigma_{2}^{2}}{1-\epsilon }\left(\kappa_{2}^{2}+\kappa_{1}^{2}+\left(\kappa_{4}^{2}+C_{f}^{2}\right)\nu^{-2}\right)]$, and $\kappa_{6}^{2}:=\kappa_{2}^{2}+\kappa_{3}^{2}\nu^{-1}+\frac{\kappa_{5}^{2}}{1-\sigma_{2}^{2}}+\frac{2}{1-\sigma_{2}^{2}}\kappa_{5}^{2}.$*

**Proof.**  Taking $\left(v,q\right)=\left(v_{h},p_{h}\right)$ in (33) and subtracting (61), we arrive at

(81) $$\begin{array}{c} \left(e_{ht}^{l},v_{h}\right)+\nu a\left(e_{h}^{l},v_{h}\right)-d\left(v_{h},\xi_{h}^{l}\right)+d\left(e_{h}^{l},q_{h}\right)+b\left(e_{h}^{l-1},u_{h}^{l},v_{h}\right)+b\left(u^{l-1},e_{h}^{l},v_{h}\right)=0, \end{array}$$

where $e_{h}^{l}:=u^{l}-u_{h}^{l}=u^{l}-R_{h}\left(u^{l},p^{l}\right)+R_{h}\left(u^{l},p^{l}\right)-u_{h}^{l}:=\eta_{h}^{l}+\theta_{h}^{l}$ and $\xi_{h}^{l}:=p^{l}-p_{h}^{l}=p^{l}-Q_{h}\left(u^{l},p^{l}\right)+Q_{h}\left(u^{l},p^{l}\right)-p_{h}^{l}:=\gamma_{h}^{l}+\zeta_{h}^{l}$. Setting $\left(v_{h},q_{h}\right)=e^{\nu \lambda_{1}t}\left(\theta_{h}^{l},\zeta_{h}^{l}\right)$ in (81) and using the Galerkin projection (65), we obtain

(82) $$\begin{array}{cc} & \frac{1}{2}\frac{d}{dt}e^{\nu \lambda_{1}t}|e_{h}^{l}{|}^{2}+\nu e^{\nu \lambda_{1}t}\left|\right|\theta_{h}^{l}{\left|\right|}^{2}+b\left(e_{h}^{l-1},u_{h}^{l},e^{\nu \lambda_{1}t}\theta_{h}^{l}\right)+b\left(u^{l-1},e_{h}^{l},e^{\nu \lambda_{1}t}\theta_{h}^{l}\right)\\ = & e^{\nu \lambda_{1}t}\left(e_{ht}^{l},\eta_{h}^{l}\right)+\frac{1}{2}\nu \lambda_{1}e^{\nu \lambda_{1}t}{\left|e_{h}^{l}\right|}^{2}. \end{array}$$

Using (3), (6), (7) and (56), it holds that

$$\begin{array}{cc} \left(e_{ht}^{l},\eta_{h}^{l}\right) & \leq |e_{ht}^{l}\left|\;\right|\eta_{h}^{l}|,\\ \frac{1}{2}\nu \lambda_{1}{\left|e_{h}^{l}\right|}^{2} & \leq \frac{1}{2}\nu \lambda_{1}\left(\right|\eta_{h}^{l}{|}^{2}+|\theta_{h}^{l}{|}^{2})\\ & \leq \frac{\nu }{2}\left|\right|\theta_{h}^{l}{\left|\right|}^{2}+\frac{1}{2}\nu \lambda_{1}{\left|\eta_{h}^{l}\right|}^{2},\\ \left|b(e_{h}^{l-1},u_{h}^{l},\theta_{h}^{l})\right| & \leq |b\left(\eta_{h}^{l-1},u_{h}^{l},\theta_{h}^{l}\right)|+|b\left(\theta_{h}^{l-1},u_{h}^{l},\theta_{h}^{l}\right)|,\\ \left|b(\eta_{h}^{l-1},u_{h}^{l},\theta_{h}^{l})\right| & \leq N||\eta_{h}^{l-1}\left||\;|\right|\theta_{h}^{l}\left||\;|\right|u_{h}^{l}\left|\right|\\ & \leq \frac{\epsilon \nu }{4}\left|\right|\theta_{h}^{l}{\left|\right|}^{2}+\frac{N^{2}}{\nu \epsilon }\left|\right|u_{h}^{l}{\left|\right|}^{2}\left|\right|\eta_{h}^{l-1}{\left|\right|}^{2},\\ \left|b(\theta_{h}^{l-1},u_{h}^{l},\theta_{h}^{l})\right| & \leq \frac{(1-\epsilon )\nu }{2}\left|\right|\theta_{h}^{l}{\left|\right|}^{2}+\frac{N^{2}}{2(1-\epsilon )\nu }|A_{h}u_{h}^{l}{|}^{2}{\left|\theta_{h}^{l-1}\right|}^{2}\\ & \leq \frac{(1-\epsilon )\nu }{2}\left|\right|\theta_{h}^{l}{\left|\right|}^{2}+\frac{N^{2}}{2(1-\epsilon )\nu }|A_{h}u_{h}^{l}{|}^{2}\left(\right|e_{h}^{l-1}{|}^{2}+|\eta_{h}^{l-1}{|}^{2}),\\ \left|b(u^{l-1},e_{h}^{l},\theta_{h}^{l})\right| & =|b(u_{h}^{l-1},\eta_{h}^{l},\theta_{h}^{l})|\\ & \leq \frac{\epsilon \nu }{4}\left|\right|\theta_{h}^{l}{\left|\right|}^{2}+\frac{N^{2}}{\nu \epsilon }\left|\right|u_{h}^{l-1}{\left|\right|}^{2}\left|\right|\eta_{h}^{l}{\left|\right|}^{2},\\ |e_{h}^{l}{\left(0\right)|}^{2} & =|u^{l}\left(0\right)-u_{h}^{l}\left(0\right)|=|u_{0}-P_{h}u_{0}{|}^{2}\leq ch^{2}\left|\right|u_{0}{\left|\right|}^{2}. \end{array}$$

Substituting these inequalities into (82); integrating from 0 to *t*; multiplying by $e^{-\nu \lambda_{1}t}$; and using Lemmas 3 and 4, (4) and (78), we obtain

(83) $$\begin{array}{cc} \;|e_{h}^{l}{|}^{2}\leq & ch^{2}\left|\right|u_{0}{\left|\right|}^{2}+\nu \lambda_{1}e^{-\nu \lambda_{1}t}\int_{0}^{t}e^{\nu \lambda_{1}s}{\left|\eta_{h}^{l}\right|}^{2}ds\\ & +ch^{2}{\left(e^{-\nu \lambda_{1}t}\int_{0}^{t}e^{\nu \lambda_{1}s}|u_{s}^{l}{|}^{2}+{\left|u_{hs}^{l}\right|}^{2}ds\right)}^{\frac{1}{2}}{\left(e^{-\nu \lambda_{1}t}\int_{0}^{t}e^{\nu \lambda_{1}s}(|Au^{l}{|}^{2}+\left|\right|p^{l}{\left|\right|}_{1}^{2}/\nu^{2})ds\right)}^{\frac{1}{2}}\\ & +\frac{2N^{2}\left|\right|u_{h}^{l}{\left|\right|}^{2}}{\nu \epsilon }e^{-\nu \lambda_{1}t}\int_{0}^{t}e^{\nu \lambda_{1}s}\left|\right|\eta_{h}^{l-1}{\left|\right|}^{2}ds+\frac{2N^{2}\left|\right|u_{h}^{l-1}{\left|\right|}^{2}}{\nu \epsilon }e^{-\nu \lambda_{1}t}\int_{0}^{t}e^{\nu \lambda_{1}s}\left|\right|\eta_{h}^{l}{\left|\right|}^{2}ds\\ & +\frac{N^{2}}{(1-\epsilon )\nu }|\eta_{h}^{l-1}{|}^{2}e^{-\nu \lambda_{1}t}\int_{0}^{t}e^{\nu \lambda_{1}s}|A_{h}u_{h}^{l}{|}^{2}ds+\frac{N^{2}}{(1-\epsilon )\nu }|e_{h}^{l-1}{|}^{2}e^{-\nu \lambda_{1}t}\int_{0}^{t}e^{\nu \lambda_{1}s}{\left|A_{h}u_{h}^{l}\right|}^{2}ds\\ \;\leq & \kappa_{5}^{2}h^{2}+\sigma_{2}^{2}{\left|e_{h}^{l-1}\right|}^{2}\\ \leq & \kappa_{5}^{2}\frac{1-\sigma_{2}^{2(l-1)}}{1-\sigma_{2}^{2}}h^{2}+\sigma_{2}^{2l}{\left|e_{h}^{0}\right|}^{2}. \end{array}$$

Letting l→+∞ in the above inequality and using Theorem 4, we obtain (79).

On the other hand, the trilinear terms in (82) can be estimated as follows:

$$\begin{array}{cc} \left|b(\eta_{h}^{l-1},u_{h}^{l},\theta_{h}^{l})\right| & =|b(\eta_{h}^{l-1},\theta_{h}^{l},u_{h}^{l})|\\ & \leq \frac{\nu }{4}\left|\right|\theta_{h}^{l}{\left|\right|}^{2}+\frac{N^{2}}{\nu }\left|\right|u_{h}^{l}{\left|\right|}^{2}\left|\right|\eta_{h}^{l-1}{\left|\right|}^{2},\\ \left|b(\theta_{h}^{l-1},u_{h}^{l},\theta_{h}^{l})\right| & \leq \frac{\nu }{4}\left|\right|\theta_{h}^{l}{\left|\right|}^{2}+\frac{N^{2}}{\nu }|A_{h}u_{h}^{l}{|}^{2}{\left|\theta_{h}^{l-1}\right|}^{2},\\ \left|b(u^{l-1},e_{h}^{l},\theta_{h}^{l})\right| & =|b(u_{h}^{l-1},\eta_{h}^{l},\theta_{h}^{l})|\\ & \leq \frac{\nu }{4}\left|\right|\theta_{h}^{l}{\left|\right|}^{2}+\frac{N^{2}}{\nu }\left|\right|u_{h}^{l-1}{\left|\right|}^{2}\left|\right|\eta_{h}^{l}{\left|\right|}^{2}. \end{array}$$

Thus, we have

$$\begin{array}{cc} & \nu e^{-\nu \lambda_{1}t}\int_{0}^{t}e^{\nu \lambda_{1}s}\left|\right|e_{h}^{l}{\left|\right|}^{2}ds\\ \leq & \nu e^{-\nu \lambda_{1}t}\int_{0}^{t}e^{\nu \lambda_{1}s}\left|\right|\eta_{h}^{l}{\left|\right|}^{2}ds+\nu e^{-\nu \lambda_{1}t}\int_{0}^{t}e^{\nu \lambda_{1}s}\left|\right|\theta_{h}^{l}{\left|\right|}^{2}ds\\ \leq & \nu e^{-\nu \lambda_{1}t}\int_{0}^{t}e^{\nu \lambda_{1}s}\left|\right|\eta_{h}^{l}{\left|\right|}^{2}ds+2\nu \lambda_{1}|e_{h}^{l}{|}^{2}e^{-\nu \lambda_{1}t}\int_{0}^{t}e^{\nu \lambda_{1}s}ds+ch^{2}\left|\right|u_{0}{\left|\right|}^{2}\\ & +4h^{2}{\left(e^{-\nu \lambda_{1}t}\int_{0}^{t}e^{\nu \lambda_{1}s}|u_{s}^{l}{|}^{2}+{\left|u_{hs}^{l}\right|}^{2}ds\right)}^{\frac{1}{2}}{\left(e^{-\nu \lambda_{1}t}\int_{0}^{t}e^{\nu \lambda_{1}s}(|Au^{l}{|}^{2}+\left|\right|p^{l}{\left|\right|}_{1}^{2}/\nu^{2})ds\right)}^{\frac{1}{2}}\\ & +\frac{4N^{2}\left|\right|u_{h}^{l}{\left|\right|}^{2}}{\nu }e^{-\nu \lambda_{1}t}\int_{0}^{t}e^{\nu \lambda_{1}s}\left|\right|\eta_{h}^{l-1}{\left|\right|}^{2}ds+\frac{4N^{2}\left|\right|u_{h}^{l-1}{\left|\right|}^{2}}{\nu }e^{-\nu \lambda_{1}t}\int_{0}^{t}e^{\nu \lambda_{1}s}\left|\right|\eta_{h}^{l}{\left|\right|}^{2}ds\\ & +\frac{4N^{2}}{\nu }|\eta_{h}^{l-1}{|}^{2}e^{-\nu \lambda_{1}t}\int_{0}^{t}e^{\nu \lambda_{1}s}|A_{h}u_{h}^{l}{|}^{2}ds+\frac{4N^{2}}{\nu }|e_{h}^{l-1}{|}^{2}e^{-\nu \lambda_{1}t}\int_{0}^{t}e^{\nu \lambda_{1}s}{\left|A_{h}u_{h}^{l}\right|}^{2}ds, \end{array}$$

which, together with Lemma 3, Theorem 4, and (79), implies (80). The proof is completed. □

**Lemma** **6.** *Under the assumptions of Lemma 5, it holds that*

(84) $$\begin{array}{cc} \tau \left(t\right)||u-u_{h}{\left|\right|}^{2} & \leq \kappa_{7}^{2}h^{2}, \end{array}$$

(85) $$\begin{array}{cc} e^{-\nu \lambda_{1}t}\int_{0}^{t}e^{\nu \lambda_{1}s}\tau \left(s\right){\left|u_{s}-u_{hs}\right|}^{2}ds & \leq \kappa_{8}^{2}h^{2}, \end{array}$$

*where $\kappa_{7}^{2}$ and $\kappa_{8}^{2}$ are power-type functions satisfying $\kappa_{7}^{2}:=\nu^{-1}\left\{\kappa_{4}^{2}\nu^{-1}+\left[1+\left(\lambda_{1}+9\right)\nu +\sqrt{\nu (1+\nu \lambda_{1})}\right]\kappa_{4}^{2}\nu^{-2}+\nu^{-1}{\tilde{C}}_{f}^{2}+\left(1+\nu \lambda_{1}+5\nu \right)\kappa_{4}^{2}+\left[1+\left(\lambda_{1}+9\right)\nu +\sqrt{\nu (1+\nu \lambda_{1})}\right]\kappa_{4}^{2}\nu^{-1}+{\tilde{C}}_{f}^{2}\nu \kappa_{2}^{2}+\kappa_{3}^{2}+N^{2}\kappa_{1}\left(\kappa_{1}+\kappa_{4}\nu^{-1}\right)\kappa_{6}^{2}+N^{2}\kappa_{1}\kappa_{2}\kappa_{5}\kappa_{6}\nu^{-1/2}\right\}$ and $\kappa_{8}^{2}:=\kappa_{7}^{2}+\nu \kappa_{1}^{2}+2\kappa_{4}^{2}+\kappa_{4}^{2}+\left[1+\left(\lambda_{1}+9\right)\nu +\sqrt{\nu (1+\nu \lambda_{1})}\right]\kappa_{4}^{2}\nu^{-2}+\nu^{-1}{\tilde{C}}_{f}^{2}$.*

**Proof.**  Taking $\left(v,q\right)=\left(v_{h},q_{h}\right)$ in (14) and subtracting (63), we have

(86) $$\begin{array}{c} \left(e_{ht},v_{h}\right)+\nu a\left(e_{h},v_{h}\right)-d\left(v_{h},\xi_{h}\right)+d\left(e_{h},q_{h}\right)+b\left(e_{h},u,v_{h}\right)+b\left(u_{h},e_{h},v_{h}\right)=0, \end{array}$$

where $e_{h}=u-u_{h}=u-R_{h}\left(u,p\right)+R_{h}\left(u,p\right)-u_{h}=\eta_{h}+\theta_{h}$ and $\xi_{h}=p-p_{h}=p-Q_{h}\left(u,p\right)+Q_{h}\left(u,p\right)-p_{h}=\gamma_{h}+\zeta_{h}$. Differentiating $d(e_{h},q_{h})$ with respect to *t*, using the Galerkin projection (65), and taking $\left(v_{h},q_{h}\right)=\tau \left(t\right)e^{\nu \lambda_{1}t}\left(\theta_{ht},\xi_{h}\right)$ in (86), we arrive at

(87) $$\begin{array}{cc} & \tau \left(t\right)e^{\nu \lambda_{1}t}|\theta_{ht}{|}^{2}+\frac{\nu }{2}\frac{d}{dt}\tau \left(t\right)e^{\nu \lambda_{1}t}\left|\right|\theta_{h}{\left|\right|}^{2}+b\left(e_{h},u,\tau \left(t\right)e^{\nu \lambda_{1}t}\theta_{ht}\right)+b\left(u_{h},e_{h},\tau \left(t\right)e^{\nu \lambda_{1}t}\theta_{ht}\right)\\ = & \tau \left(t\right)e^{\nu \lambda_{1}t}\left(\eta_{ht},\theta_{ht}\right)+\frac{\nu }{2}e^{\nu \lambda_{1}t}\left|\right|\theta_{h}{\left|\right|}^{2}+\frac{\nu }{2}\tau \left(t\right)\nu \lambda_{1}e^{\nu \lambda_{1}t}\left|\right|\theta_{h}{\left|\right|}^{2}. \end{array}$$

Since

$$\begin{array}{cc} \left|\tau \left(t\right)(\eta_{ht},\tau \left(t\right)\theta_{ht})\right| & \leq \frac{1}{4}\tau \left(t\right)|\theta_{ht}{|}^{2}+\tau \left(t\right){\left|\eta_{ht}\right|}^{2},\\ \left|b(e_{h},u_{h}^{l},\tau \left(t\right)\theta_{ht})\right| & \leq \frac{1}{8}\tau \left(t\right)|\theta_{ht}{|}^{2}+2N^{2}\tau \left(t\right)||u_{h}\left||\;\right|A_{h}u_{h}\left|\;\right|e_{h}\left|\;|\right|e_{h}\left|\right|,\\ \left|b(u,e_{h},\tau \left(t\right)\theta_{ht})\right| & \leq \frac{1}{8}\tau \left(t\right)|\theta_{ht}{|}^{2}+2N^{2}\tau \left(t\right)|u|\;|Au|\;||e_{h}{\left|\right|}^{2}, \end{array}$$

putting these inequalities into (87), integrating from 0 to *t*, multiplying by $e^{-\nu \lambda_{1}t}$, and using Lemmas 3 and 5, we have

$$\begin{array}{cc} & \nu \tau \left(t\right)||\theta_{h}{\left|\right|}^{2}+e^{-\nu \lambda_{1}t}\int_{0}^{t}e^{\nu \lambda_{1}s}\tau \left(s\right){\left|\theta_{hs}\right|}^{2}ds\\ \leq & 2e^{-\nu \lambda_{1}t}\int_{0}^{t}e^{\nu \lambda_{1}s}\tau \left(s\right){\left|\eta_{hs}^{l}\right|}^{2}ds\\ & +\nu e^{-\nu \lambda_{1}t}\int_{0}^{t}e^{\nu \lambda_{1}s}\left(|\right|e_{h}{\left|\right|}^{2}+\left|\right|\eta_{h}{\left|\right|}^{2})ds+\nu^{2}\lambda_{1}e^{-\nu \lambda_{1}t}\int_{0}^{t}e^{\nu \lambda_{1}s}\left(|\right|e_{h}{\left|\right|}^{2}+\left|\right|\eta_{h}{\left|\right|}^{2})ds\\ & +4N^{2}\left|u|\;|Au\right|e^{-\nu \lambda_{1}t}\int_{0}^{t}e^{\nu \lambda_{1}s}\left|\right|e_{h}{\left|\right|}^{2}ds\\ & +4N^{2}\left|\right|u_{h}\left||\;\right|e_{h}|{\left(e^{-\nu \lambda_{1}t}\int_{0}^{t}e^{\nu \lambda_{1}s}{\left|A_{h}u_{h}\right|}^{2}ds\right)}^{1/2}{\left(e^{-\nu \lambda_{1}t}\int_{0}^{t}e^{\nu \lambda_{1}s}\left|\right|e_{h}{\left|\right|}^{2}ds\right)}^{1/2}\\ \leq & ch^{2}e^{-\nu \lambda_{1}t}\int_{0}^{t}e^{\nu \lambda_{1}s}\tau \left(s\right)(||u_{t}{\left|\right|}^{2}+\left|\right|p_{t}{\left|\right|}_{1}^{2}/\nu^{2})ds+ch^{2}e^{-\nu \lambda_{1}t}\int_{0}^{t}e^{\nu \lambda_{1}s}\tau \left(s\right)(\nu ||u_{s}{\left|\right|}_{2}^{2}+\left|\right|p_{s}{\left|\right|}_{1}^{2}/\nu )ds\\ & +ch^{2}e^{-\nu \lambda_{1}t}\int_{0}^{t}e^{\nu \lambda_{1}s}(\nu^{2}{\left||u|\right|}_{2}^{2}+{\left||p|\right|}_{1}^{2})ds\\ & +4N^{2}\left|u|\;|Au\right|e^{-\nu \lambda_{1}t}\int_{0}^{t}e^{-\nu \lambda_{1}s}\left|\right|e_{h}{\left|\right|}^{2}ds\\ & +4N^{2}\left|\right|u_{h}\left||\;\right|e_{h}|{\left(e^{-\nu \lambda_{1}t}\int_{0}^{t}e^{\nu \lambda_{1}s}{\left|A_{h}u_{h}\right|}^{2}ds\right)}^{1/2}{\left(e^{-\nu \lambda_{1}t}\int_{0}^{t}e^{\nu \lambda_{1}s}\left|\right|e_{h}{\left|\right|}^{2}ds\right)}^{1/2}, \end{array}$$

which, together with Theorems 2–4 and Lemmas 3 and 5 yields Lemma 6. □

**Lemma** **7.** *Under the assumptions of Lemma 5, it holds that*

(88) $$\begin{array}{cc} \tau^{2}\left(t\right){\left|u_{t}-u_{ht}\right|}^{2} & \leq \kappa_{9}^{2}h^{2}, \end{array}$$

(89) $$\begin{array}{cc} e^{-\nu \lambda_{1}t}\int_{0}^{t}\tau^{2}\left(s\right)e^{\nu \lambda_{1}s}\left|\right|u_{s}-u_{hs}{\left|\right|}^{2}ds & \leq \kappa_{10}^{2}h^{2}, \end{array}$$

*where $\kappa_{9}^{2}$ and $\kappa_{10}^{2}$ are power-type functions satisfying $\kappa_{9}^{2}:=c\nu^{-1}\{{\left[1+\left(\lambda_{1}+9\right)\nu +\sqrt{\nu (1+\nu \lambda_{1})}\right]}^{1/2}$*

*$\left[\left(1+\nu \lambda_{1}\right)\nu^{-2}+5\nu^{-1}+{\left[1+\left(\lambda_{1}+9\right)\nu +\sqrt{\nu (1+\nu \lambda_{1})}\right]}^{1/2}\nu^{-1}\right]\kappa_{4}^{2}+\nu {\tilde{C}}_{f}^{2}+\kappa_{8}^{2}+N^{2}\kappa_{1}\kappa_{4}\kappa_{8}^{2}\nu^{-1}+\frac{1}{\sqrt{1-\sigma_{2}^{2}}}N^{2}\kappa_{1}^{2}\kappa_{5}\nu -1/2\}$ and* *$\kappa_{10}^{2}:=\kappa_{9}^{2}+\left(1+\nu \lambda_{1}+5\nu \right)\kappa_{4}^{2}+\left[1+\left(\lambda_{1}+9\right)\nu +\sqrt{\nu (1+\nu \lambda_{1})}\right]\kappa_{4}^{2}\nu^{-1}+{\tilde{C}}_{f}^{2}$.*

**Proof.**  Differentiating (86) with respect to *t*, using the Galerkin projection (65), and taking $\left(v_{h},q_{h}\right)=\tau^{2}\left(t\right)e^{\nu \lambda_{1}t}\left(\theta_{ht},\xi_{ht}\right)$, we arrive at

(90) $$\begin{array}{cc} & \frac{1}{2}\frac{d}{dt}\tau^{2}\left(t\right)e^{\nu \lambda_{1}t}|e_{ht}{|}^{2}+\nu \tau^{2}\left(t\right)||\theta_{ht}{\left|\right|}^{2}+e^{\nu \lambda_{1}t}b\left(e_{ht},u,\tau^{2}\left(t\right)\theta_{ht}\right)+e^{\nu \lambda_{1}t}b\left(e_{h},u_{t},\tau^{2}\left(t\right)\theta_{ht}\right)\\ & +e^{\nu \lambda_{1}t}b\left(u_{ht},e_{h}^{l},\tau^{2}\left(t\right)\theta_{ht}\right)+e^{\nu \lambda_{1}t}b\left(u_{h},e_{ht},\tau^{2}\left(t\right)\theta_{ht}\right)\\ = & e^{\nu \lambda_{1}t}\left(e_{htt},\tau^{2}\left(t\right)\eta_{ht}\right)+\tau \left(t\right)e^{\nu \lambda_{1}t}|e_{ht}{|}^{2}+\frac{1}{2}\nu \lambda_{1}\tau^{2}\left(t\right)e^{\nu \lambda_{1}t}{\left|e_{ht}\right|}^{2}. \end{array}$$

Since

$$\begin{array}{cc} \left|(e_{htt},\tau^{2}\left(t\right)\eta_{ht})\right| & \leq \tau^{2}\left(t\right)|e_{htt}\left|\;\right|\eta_{ht}|,\\ \left|b(e_{ht},u,\tau^{2}\left(t\right)\theta_{ht})\right| & \leq \frac{\nu }{8}\tau^{2}\left(t\right)||\theta_{ht}{\left|\right|}^{2}+2N^{2}\tau^{2}\left(t\right)|u|\;|Au|\;|e_{ht}{|}^{2},\\ \left|b(e_{h},u_{t},\tau^{2}\left(t\right)\theta_{ht})\right| & \leq \frac{\nu }{8}\tau^{2}\left(t\right)||\theta_{ht}{\left|\right|}^{2}+2N^{2}\tau^{2}\left(t\right)|u_{t}\left|\;|\right|u_{t}\left||\;\right|e_{h}\left|\;|\right|e_{h}\left|\right|,\\ \left|b(u_{ht},e_{h},\tau^{2}\left(t\right)\theta_{ht})\right| & \leq \frac{\nu }{8}\tau^{2}\left(t\right)||\theta_{ht}{\left|\right|}^{2}+2N^{2}\tau^{2}\left(t\right)|u_{ht}\left|\;|\right|u_{ht}\left||\;\right|e_{h}\left|\;|\right|e_{h}\left|\right|,\\ \left|b(u_{h},e_{ht},\tau^{2}\left(t\right)\theta_{ht})\right| & \leq \frac{\nu }{8}\tau^{2}\left(t\right)||\theta_{ht}{\left|\right|}^{2}+2N^{2}\tau^{2}\left(t\right)|u_{h}\left|\;\right|A_{h}u_{h}\left|\;\right|e_{ht}{|}^{2}, \end{array}$$

putting these inequalities into (90), integrating from 0 to *t*, multiplying by $e^{-\nu \lambda_{1}t}$, and using Theorems 2–4, we have

$$\begin{array}{cc} & \tau^{2}\left(t\right)|e_{ht}{|}^{2}+\nu e^{-\nu \lambda_{1}t}\int_{0}^{t}e^{\nu \lambda_{1}s}\tau^{2}\left(s\right)||\theta_{hs}^{l}{\left|\right|}^{2}ds\\ \leq & 2{\left(e^{-\nu \lambda_{1}t}\int_{0}^{t}e^{\nu \lambda_{1}s}\tau \left(s\right)(|u_{ss}{|}^{2}+|u_{hss}{|}^{2})ds\right)}^{\frac{1}{2}}{\left(e^{-\nu \lambda_{1}t}\int_{0}^{t}e^{\nu \lambda_{1}s}\tau \left(s\right){\left|\eta_{hs}\right|}^{2}ds\right)}^{\frac{1}{2}}\\ & +\left(2+\nu \lambda_{1}\right)e^{-\nu \lambda_{1}t}\int_{0}^{t}e^{\nu \lambda_{1}s}\tau \left(s\right)|e_{hs}{|}^{2}ds+4N^{2}\left|u|\;|Au\right|e^{-\nu \lambda_{1}t}\int_{0}^{t}e^{\nu \lambda_{1}s}\tau \left(s\right){\left|e_{hs}\right|}^{2}ds\\ & +4N^{2}|u_{h}\left|\;\right|A_{h}u_{h}|e^{-\nu \lambda_{1}t}\int_{0}^{t}e^{\nu \lambda_{1}s}\tau \left(s\right){\left|e_{hs}\right|}^{2}ds\\ & +4N^{2}\tau \left(t\right)|u_{t}\left|\;\right|e_{h}|{\left(e^{-\nu \lambda_{1}t}\int_{0}^{t}e^{\nu \lambda_{1}s}\tau \left(s\right)||u_{s}{\left|\right|}^{2}ds\right)}^{\frac{1}{2}}{\left(e^{-\nu \lambda_{1}t}\int_{0}^{t}e^{\nu \lambda_{1}s}\tau \left(s\right)||e_{h}{\left|\right|}^{2}ds\right)}^{\frac{1}{2}}\\ & +4N^{2}\tau \left(t\right)|u_{ht}\left|\;\right|e_{h}|{\left(e^{-\nu \lambda_{1}t}\int_{0}^{t}e^{\nu \lambda_{1}s}\tau \left(s\right)||u_{hs}{\left|\right|}^{2}ds\right)}^{\frac{1}{2}}{\left(e^{-\nu \lambda_{1}t}\int_{0}^{t}e^{\nu \lambda_{1}s}\tau \left(s\right)||e_{h}{\left|\right|}^{2}ds\right)}^{\frac{1}{2}}. \end{array}$$

Using the triangle inequality and Lemmas 3, 5, and 6, we can derive (88) and (89). The proof is completed. □

**Lemma** **8.** *Under the assumptions of Lemma 5, it holds that*

(91) $$\begin{array}{c} \tau^{2}\left(t\right){\left|p-p_{h}\right|}^{2}\leq \kappa_{11}^{2}h^{2}, \end{array}$$

*where $\kappa_{11}^{2}$ is a power-type function satisfying* *$\kappa_{11}^{2}:=\beta_{h}^{-1}\left[\kappa_{9}^{2}+\kappa_{7}^{2}+\frac{1}{1-\sigma_{2}^{2}}N\kappa_{5}\kappa_{7}\kappa_{2}{\left(\nu^{2}\kappa_{1}^{2}+2\kappa_{4}^{2}\right)}^{1/2}\nu^{-1}+N\kappa_{7}^{2}\kappa_{1}{\left(\nu^{2}\kappa_{1}^{2}+2\kappa_{4}^{2}\right)}^{1/2}\nu^{-1}\right]$.*

**Proof.**  Using (60) and (86), we obtain

$$\begin{array}{cc} \beta_{h}\left|p-p_{h}\right|\leq & \underset{v_{h}\in X,v_{h}\neq 0}{\sup}\frac{\left|d(v_{h},\xi_{h})\right|}{\left|\right|v_{h}\left|\right|}\\ \leq & \frac{|\left(e_{ht},v_{h}\right)+\nu a\left(e_{h},v_{h}\right)+b\left(e_{h},u,v_{h}\right)+b\left(u_{h},e_{h},v_{h}\right)|}{\left|\right|v_{h}\left|\right|}\\ \leq & |e_{ht}\left|+\nu |\right|e_{h}\left||+N\right|e_{h}{|}^{1/2}\left|\right|e_{h}{\left|\right|}^{1/2}{\left||u|\right|}^{1/2}{\left|Au\right|}^{1/2}+N|u_{h}{|}^{1/2}|A_{h}u_{h}{|}^{1/2}\left|\right|e_{h}\left|\right|, \end{array}$$

which, together with Lemmas 5–7 and Theorems 2–4, yields (91). The proof is completed. □

From Lemmas 5, 6, and 8, we arrive at the main result of this paper as follows.

**Theorem** **5.** *Assuming that (* ***A1*** *)–(* ***A3*** *) and (78) hold, and (u,p) and $\left(u_{h},p_{h}\right)$ are the solutions of (14) and (15) and (63) and (64), respectively, then it holds that*

$$\begin{array}{cc} |u-u_{h}{|}^{2} & \leq \frac{\kappa_{5}^{2}}{1-\sigma_{2}^{2}}h^{2},\\ \tau \left(t\right)||u-u_{h}{\left|\right|}^{2} & \leq \kappa_{7}^{2}h^{2},\\ \tau^{2}\left(t\right){\left|p-p_{h}\right|}^{2} & \leq \kappa_{11}^{2}h^{2}. \end{array}$$

## 5. Numerical Examples

In this section, we will show some numerical examples to confirm the theoretical predictions derived above.

**Figure 1:**
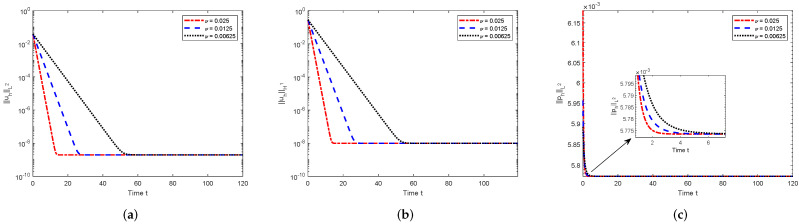
Numerical solutions with respect to the time *t* (steady body force). (**a**) $\left|\right|u_{h}{\left|\right|}_{L^{2}}$; (**b**) $\left|\right|u_{h}{\left|\right|}_{H^{1}}$; (**c**) $\left|\right|p_{h}{\left|\right|}_{L^{2}}$.

Let the domain Ω=(0,1)×(0,1); the spatial mesh h=1/30; the initial data $u_{0}={\left(u_{1}\left(0\right),u_{2}\left(0\right)\right)}^{T}={\left(10x_{1}^{2}{\left(x_{1}-1\right)}^{2}x_{2}\left(x_{2}-1\right)\left(2x_{2}-1\right),-10x_{1}\left(x_{1}-1\right)\left(2x_{1}-1\right)x_{2}^{2}{\left(x_{2}-1\right)}^{2}\right)}^{T}$; the simulation time T=120; and ν=1/40, 1/80, and 1/160, respectively. Due to the constants *N* and $\lambda_{1}$ only depending on the domain, for the computational domain considered here, it holds that N≤1/2π and ${\left||f|\right|}_{-1}\leq \left|f\right|/\left(\sqrt{2}\pi \right)$ (see [32]). Thus, it is easy to check that the assumption (21) is valid in this case. Firstly, setting the body force $f={\left(f_{1},f_{2}\right)}^{T}={\left(0.01,0.01\right)}^{T}$, we investigate the development of the numerical solutions with respect to the time *t*. From the definitions of $\kappa_{1}$ and $\kappa_{2}$, we know that with this given body force independent of the time here, the numerical solutions will decay as an exponential function with respect to the time *t* and arrive at a steady state when the time *t* is big enough; the smaller the viscosity is, the bigger the numerical solution will be. Furthermore, the smaller the viscosity is, the slower the system will decay. All of these are consistent with the results shown in Figure 1.

**Figure 2:**
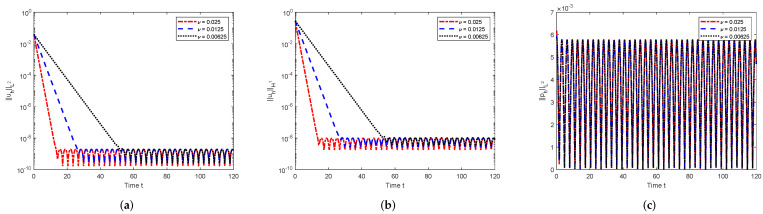
Numerical solutions with respect to the time *t* (periodic body force). (**a**) $\left|\right|u_{h}{\left|\right|}_{L^{2}}$; (**b**) $\left|\right|u_{h}{\left|\right|}_{H^{1}}$; (**c**) $\left|\right|p_{h}{\left|\right|}_{L^{2}}$.

Secondly, with a periodic body force $f={\left(f_{1},f_{2}\right)}^{T}={\left(0.01\cos\left(t\right),0.01\cos\left(t\right)\right)}^{T}$ and the other computational parameters the same as that in the above, we collect the numerical results in Figure 2. We can see that the performance is similar to that for the steady body force, except the system will arrive at the periodic state as the time develops. The reason is that the exponential terms decay fast in $\kappa_{1}$ and $\kappa_{2}$ as *t* increases, and the periodic function cos(t) will be dominant after some critical times (these times depend on the viscosity and the norm investigated). These confirm the theoretical analysis again.

**Figure 3:**
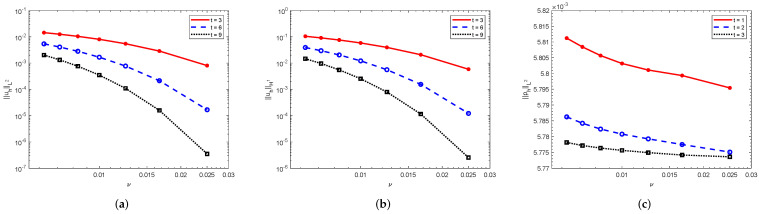
Numerical solutions with respect to the viscosity ν. (**a**) $\left|\right|u_{h}{\left|\right|}_{L^{2}}$; (**b**) $\left|\right|u_{h}{\left|\right|}_{H^{1}}$; (**c**) $\left|\right|p_{h}{\left|\right|}_{L^{2}}$.

Finally, we study the relationship between the numerical solutions and the viscosity. With ν=1/40, 1/60, 1/80, 1/100, 1/120, 1/140, 1/160, respectively, we plot the development of the approximation in Figure 3. The graphs imply that the numerical solutions increase as a power function with respect to the viscosity, not as an exponential function. All of these suggest that the analysis in this paper is sharper than that in the references.

## 6. Conclusions

We derive finite element error estimates with power-type asymptotic constants for long-time approximation of the Navier–Stokes equations, which can describe the approach feature better compared with ones with exponential-type asymptotic coefficients errors. The main technique used in this paper is to construct a kind of fixed-point operator, by which the Gronwall lemma is avoided in the analysis. The analysis is confirmed by some numerical examples. This idea can be extended to the fully discrete and other time-dependent problems, which will be considered in our future work.

## Data Availability

Not applicable.
